# Three-dimensional Zn-based alloys for dendrite-free aqueous Zn battery in dual-cation electrolytes

**DOI:** 10.1038/s41467-022-35618-2

**Published:** 2022-12-23

**Authors:** Huajun Tian, Guangxia Feng, Qi Wang, Zhao Li, Wei Zhang, Marcos Lucero, Zhenxing Feng, Zi-Le Wang, Yuning Zhang, Cheng Zhen, Meng Gu, Xiaonan Shan, Yang Yang

**Affiliations:** 1 Key Laboratory of Power Station Energy Transfer Conversion and System (North China Electric Power University), Ministry of Education, North China Electric Power University, Beijing, 102206 China; 2 NanoScience Technology Center, University of Central Florida, Orlando, FL 32826 USA; 3 Electrical and Computer Engineering Department, University of Houston, W306, Engineering Building 2, Houston, TX 77204 USA; 4 Department of Materials Science and Engineering, Southern University of Science and Technology, Shenzhen, 518055 China; 5 Department of Materials Science and Engineering, University of Central Florida, Orlando, FL 32826 USA; 6 School of Chemical, Biological, and Environmental Engineering, Oregon State University, Corvallis, OR 97331 USA; 7 Renewable Energy and Chemical Transformation Cluster, University of Central Florida, Orlando, FL 32826 USA; 8 Department of Chemistry, University of Central Florida, Orlando, FL 32826 USA; 9 The Stephen W. Hawking Center for Microgravity Research and Education, University of Central Florida, Orlando, FL 32826 USA

**Keywords:** Batteries, Batteries

## Abstract

Aqueous zinc-ion batteries, in terms of integration with high safety, environmental benignity, and low cost, have attracted much attention for powering electronic devices and storage systems. However, the interface instability issues at the Zn anode caused by detrimental side reactions such as dendrite growth, hydrogen evolution, and metal corrosion at the solid (anode)/liquid (electrolyte) interface impede their practical applications in the fields requiring long-term performance persistence. Despite the rapid progress in suppressing the side reactions at the materials interface, the mechanism of ion storage and dendrite formation in practical aqueous zinc-ion batteries with dual-cation aqueous electrolytes is still unclear. Herein, we design an interface material consisting of forest-like three-dimensional zinc-copper alloy with engineered surfaces to explore the Zn plating/stripping mode in dual-cation electrolytes. The three-dimensional nanostructured surface of zinc-copper alloy is demonstrated to be in favor of effectively regulating the reaction kinetics of Zn plating/stripping processes. The developed interface materials suppress the dendrite growth on the anode surface towards high-performance persistent aqueous zinc-ion batteries in the aqueous electrolytes containing single and dual cations. This work remarkably enhances the fundamental understanding of dual-cation intercalation chemistry in aqueous electrochemical systems and provides a guide for exploring high-performance aqueous zinc-ion batteries and beyond.

## Abstract (web summary)

The dual-cations electrochemical system was considered to be a promising strategy to facilitate sluggish diffusion kinetics. Here the authors prepare zinc-based alloy anode with three-dimensional interface, thus to improve the interfacial stability, achieve high-performing battery system in the aqueous electrolytes containing dual cations.

## Introduction

Lithium-ion batteries (LIBs) with organic electrolytes have been dominating the present electric vehicles and portable devices landscape^1^. Conventional organic electrolytes for LIBs are volatile and highly flammable, which will exacerbate thermal runaway, exposure to high temperatures (> 150 °C), and eventually catching fire if experiencing short circuits, overcharging, and other thermal abuse conditions in battery operation^2,3^. In addition, LIBs deliver limited specific power capability that is partially restricted by the lower ionic conductivities (10^−2^~10^−3^ S cm^−1^, normally) of the organic electrolyte compared with aqueous electrolyte (10^−1^~10^−2^ S cm^−1^) at room temperature^4,5^. Besides, the production cost of LIBs is comparatively high, which origins from the rigorous manufacturing processes, and the high cost of Li salts and organic solvents. Particularly, the proliferation of the increasing demand for large-scale electrochemical energy storage implementations has raised concerns, including the high energy density, high safety, environmentally benignity, low production cost, and durable stability^6^. Therefore, developing novel, safe, low-cost, and durable battery systems alternatives to traditional LIBs becomes increasingly urgent and promising to satisfy the increasing demands of advanced and high-safety energy storage^7,8^.

In recent years, rechargeable aqueous metal-ion batteries (RAMBs) have been considered to be one of the most promising candidates due to the high safety of aqueous electrolytes, the high theoretical capacity of the metal anode, and the low cost^9^. However, the development of state-of-the-art RAMBs is severely plagued by the inherent instability issues at the anode/electrolyte interface, such as dendrite growth, hydrogen evolution, and metal corrosion, which hamper their practical applications in high-performance persistent energy storage systems. The exploration and optimization of RAMBs are highly dependent on improving both the thermodynamics and reaction kinetics of the metal anode. Among the RAMBs systems, aqueous Zn metal batteries (AZMBs) have gained much attention due to their relatively low cost and high energy density^10,11^. Metal Zn has low toxicity, low cost (ca. 2 USD kg^−1^), high theoretical capacity (820 mAh g^−1^ or 5854 mAh cm^−3^), much low redox potential (−0.76 V versus the standard hydrogen electrode (SHE)), high water compatibility, and intrinsic safety^12,13^. Therefore, AZMBs have been regarded as one of the most promising next-generation electrochemical storage systems^14,15^. As for AZMBs, the strategies for minimizing the side reactions concerning the dendritic growth and corrosion of Zn, as well as hydrogen evolution, have been investigated in recent years. The main strategies include surface modification^16,17^, electrolytes optimization^18^, nanostructure engineering^19^, and so forth^20^. However, the understanding and development of state-of-the-art AZIBs are still far away from practical applications. On the other hand, to achieve enhanced electrochemical performance in rechargeable aqueous batteries, many efforts have been devoted to exploring new aqueous battery chemistries based on Li^+^^21^, Na^+^^22^, K^+^^23^, Mn^2+^ ^24,25^, Mg^2+^ ^26^, Zn^2+^ ^11^, Al^3+^ ^27^, and dual-cations^28^. Compared with aqueous batteries containing a single cation, the dual-cation battery has been considered to be a promising strategy that can facilitate sluggish diffusion kinetics by adding secondary cations to the single-cation electrolytes. Meanwhile, the aqueous dual-cation batteries may increase the operating voltage and broaden the choice of electrode materials with high capacity and excellent intercalation dynamics. Dual-cation electrolytes-based aqueous batteries provide a novel method to improve the electrochemical properties of aqueous batteries through the synergy effects between various active cations. Understanding these impressive synergistic effects and mechanisms requires more fundamental research, theoretical calculations, and in-depth experiments to promote the development of dual-cation batteries with high power energy densities, long-term cycling stability, and low cost. However, the mechanism of dual-cation intercalation for aqueous batteries is still not fully understood yet.

Herein, we propose an interface material consisting of three-dimensional (3D) nanostructured Zn-Cu alloy (i.e., Zn_5_Cu), which can successfully solve the inherent interface instability issues of AZIBs by the adoption of the dual-cation (Zn^2+^/Mg^2+^ and Zn^2+^/Na^+^) electrolytes. We also suggest an approach to surface engineering the forest-like 3D Zn-Cu anode by naturally forming a thin ZnO layer to protect the Zn_5_Cu anode, which can significantly improve the electrochemical performance of AZIBs. Further, we designed an in-situ visualized protocol that can explore the metal plating/stripping dynamics under conditions analogous to the actual electrochemical environments of dual-cation battery systems. We in-situ visualize and understand the electrochemical Zn plating/stripping processes at low current densities and high current densities up to 50 mA cm^−2^ in the dual-cation electrolytes. Using the in-situ optical microscopy, we confirm that the Zn plating/stripping processes preferentially happen inside the forest-like 3D structure of the Zn-Cu anode, therefore, preventing the dendritic growth of Zn. Compared with the reported method, our 3D alloy preparation process can be performed at room temperature without any calcination treatment and also prepared in an environmentally-friendly aqueous solution for a very short reaction time (tens of minutes), which can enable the possibility of large energy storage batteries in terms of cost-effectiveness and mass-production. The paper paves the way to discover new electrochemical storage chemistry for aqueous batteries and beyond.

## Results

### Preparation and characterization of 3D Alloys

**Fig. 1:**
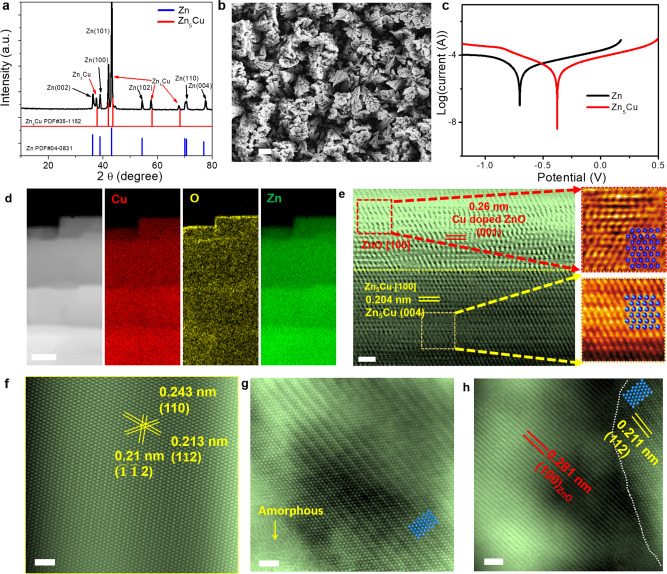
Structural characterizations of Zn-Cu anode. **a** XRD pattern. **b** SEM image of 3D Zn-Cu anodes. Scale bar: 10.0 µm. **c** Tafel plots of Zn and Zn-Cu anodes. **d** EDS-mapping of Zn_5_Cu. Scale bar: 50 nm. **e** STEM image of the ZnO/Zn_5_Cu interface. Scale bar: 1.0 nm. **f**–**h** HAADF-STEM images of the cycled Zn_5_Cu after Zn plating. Scale bar: 1.0 nm.

An alloy electrodeposition approach was developed to synthesize the proposed 3D Zn-Cu anode on the surface of the Zn substrate (See the detailed procedure in the methods section). Compared with the reported methods^29–31^, our 3D alloy preparation process can be conducted at room temperature without any calcination treatment and in an environmental-friendliness aqueous solution for a very short reaction time (tens of minutes)^17,31–34^. Figure 1a shows the X-ray diffraction (XRD) pattern of the as-prepared Zn-Cu anode, which confirms the formation of the Zn_5_Cu phase on the Zn substrate (Note: Zn-Cu alloy and Zn_5_Cu denote the same material in the following discussion). By modifying the precursors and prepared conditions in alloy electrodeposition processes, the Zn-Bi and Zn-Ni alloys can also be obtained (Supplementary Fig. 1). The forest-like 3D micro/nanostructured morphology of the Zn-Cu anode was investigated by scanning electron microscope (SEM) images as illustrated in Fig. 1b. The morphologies of Zn-Cu alloy can be largely changed in different preparation conditions via this alloy electrodeposition method as shown in Supplementary Figs. 2, 3. We adjusted the preparation condition, especially the alloy electrodeposition time from 0.5 h to 2.5 h. The morphologies of as-prepared Zn-Cu alloy can be largely changed, which would influence the electrochemical behavior of the Zn plating/stripping for aqueous Zn batteries. We have modified the ratio of two metal components in Zn-Cu alloy by tuning the preparation condition of alloy electrodeposition, such as changing the content of Cu-based precursors (CuSO_4_ salts). We found that with the increase of the content of CuSO_4_ in the alloy electrodeposition process, the as-prepared Zn-Cu alloy contains the CuZn_2_ phase **(**Supplementary Fig. 4). Meanwhile, in the alloy electrodeposition preparation process, continuous bubbles were released at the interface of a zinc foil which was dissolved in the prepared electrolyte. A thin ZnO layer on the Zn-Cu alloy anode during the alloy electrodeposition could be formed (See methods section). It has been suggested that alloyed materials can deliver improved anti-corrosive abilities compared with single-component metal materials^35^. On the other hand, the hydrogen evolution reaction can also be retarded by the ZnO architecture coating layer for aqueous Zn batteries^36^. The superior HER-inhibiting ability of the ZnO coating layer was attributed to that the layer can prevent direct contact between the aqueous solution and metal Zn anode, and therefore suppressing the side reaction^36^. To demonstrate the anti-corrosion ability of the Zn-Cu anode, we tested the Tafel polarization curves in a three-electrode system (Fig. 1c, Zn-Cu as a working electrode; Ag/AgCl as a reference, and carbon as a counter electrode). A more positively shifted corrosion potential of Zn-Cu demonstrates less corrosion tendency than the pristine Zn^37,38^. The in-situ gas analysis was tested under I-t stability measurement as shown in Supplementary Fig. 5. The current density corresponding to hydrogen evolution for Zn-Cu alloy without a ZnO layer is higher when compared with that of one with a ZnO layer. It suggests that a thin ZnO as a protective layer plays an important role in inhibiting electrode corrosion. The SEM images in Supplementary Fig. 6a–d show that Zn-Cu alloy with a ZnO layer maintains the 3D morphology after stability measurement when compared with that without an oxide layer. Additionally, the energy-dispersive X-ray spectrometry (EDS) mapping images (Supplementary Fig. 6e) of Zn-Cu alloy with a ZnO layer after stability measurement and pristine Zn substrate (Supplementary Fig. 6f) confirm the uniform distribution of Zn, Cu, and O. It further proves that the 3D morphology of Zn-Cu alloy with a ZnO layer helps to keep the structure stable and prevents the electrode etching caused by hydrogen evolution reaction (HER). Density functional theory (DFT) calculations were also employed to understand the role of Cu in Zn-Cu alloy in regulating Zn nucleation and growth in the plating process (Supplementary Fig. 7). The calculated binding energy of Zn atoms on the ZnO and Zn-Cu alloy (such as Zn_5_Cu, Zn_3_Cu_,_ or Zn_8_Cu_5_) is much higher than that of Zn, indicating that the ZnO and Zn-Cu alloy layer could be an ideal matrix to guide the Zn^2+^ plating. In contrast, the pristine Zn shows a weak interaction between Zn and Zn, resulting in a greater tendency for dendrite growth. Therefore, the Zn-Cu alloy with a ZnO layer would be a promising candidate for AZIBs due to the improved anti-corrosion property.

Aberration-corrected scanning transmission electron microscopy (STEM) was employed to study the forest-like structure of Zn-Cu, which was composed of evenly distributed Zn and Cu with an atomic ratio of 5:1 as identified by EDS mapping in Supplementary Fig. 8. The terraced branches were covered with a Zn and O-rich layer with straight interfaces (Fig. 1d and Supplementary Fig. 9). The lattice spacings of 0.260 nm and 0.204 nm were identified on the surface and the matrix of the materials, matching well with the (001) plane of ZnO and (004) plane of Zn_5_Cu, respectively (Fig. 1e). For EDS analysis of each pixel, the Zn and Cu ratio was revealed by the area of characterized K peak. To increase the S/N ratio, we integrate the EDS spectrum of the surface layer and matrix, as shown in Supplementary Fig. 10. By quantitatively calculating the K peak area of Cu and Zn, we can acquire the Zn: Cu ratio of both surface and matrix. EDS analysis reveals a 5.8 wt.% Cu-doping in the surface layer, which is lower than the Zn: Cu atomic ratio of 5:1 detected in the Zn_5_Cu matrix (Supplementary Fig. 10). Due to the similar atomic radius of Zn and Cu, the slight Cu-doping does not significantly change the lattice parameter of ZnO.

The zone axis of both the surface layer and matrix were along the [100] zone (Fig. 1e), further confirming the layer to be a ZnO shell growing epitaxially on the Zn_5_Cu core. High-angle annular dark-field detector (HAADF)-STEM images in Fig. 1f show the (110) and (112) planes of Zn_5_Cu, which agree well with the XRD results. Remarkably, the cycled Zn-Cu anode after Zn plating (denoted as Zn-Zn_5_Cu) remains the forest-like structure with an evenly distributed Zn and Cu at Zn: Cu atomic ratio of about 5:1 (Supplementary Fig. 11). The atomic arrangement of the forest-like core in the cycled Zn-Cu anode shows the same space group of *Cmmm* (Fig. 1g) compared to the Zn-Cu anode before cycling (Supplementary Fig. 12). However, the original straight interface (white dotted line in Fig. 1e) between the surface ZnO layer and the Zn_5_Cu matrix turned into a curved interface (white dotted line in Fig. 1h), indicating the atomic diffusion process occurring through the interface during the Zn plating. Moreover, the surface ZnO layer partially transformed into an amorphous structure after electrochemical cycling as labeled in Fig. 1g. Besides, the lattice spacing of the (112) plane remains 2.1 Å, indicating the core lattice constant has not been significantly affected by cycling treatment. The wettability of the Zn-Cu anode was tested and compared with the pristine Zn electrode to analyze the solid/liquid interface formation affected by the proposed interface engineering. On the more permeable surface of the Zn-Cu anode, a much smaller contact angle of 59.2 ± 0.1° was observed than the pristine Zn electrode (Supplementary Fig. 13), due to the nano capillary force of the 3D forest-like structure^39^. On the other hand, the surface composition of the Zn-Cu anode (oxygenating surface) also contributes to the improved wettability^40^, which is beneficial to the electrochemical reactions at the solid/liquid interface^41^.

We have performed X-ray absorption spectroscopy (XAS) measurements at Zn and Cu K-edge to better characterize the Zn-Cu alloy-based anode at different states of charge (SOC)^42–44^. The Zn K-edge X-ray absorption near edge structure (XANES) in Supplementary Fig. 14a reveals the slight oxidation of the Zn_5_Cu anode at a fully charged state in the dual-cation electrolytes due to the shift of XANES edge to higher energy. Additionally, we see changes in the local structure obtained. Extended X-ray absorption fine structure (EXAFS) in Supplementary Fig. 14b reveals the formation of significant Zn-O bonding (R_Zn-O_~1.5 Å) after charging. Considering the bulk sensitivity of XAS, this suggests the diffusion of ZnO layers and is consistent with STEM results. Comparing the EXAFS of the pristine and fully charged Zn_5_Cu anodes, we see an increase in the Zn-Zn scattering amplitude and a shift of the Zn-Zn peak to a higher radial distance, suggesting an increase in coordination number due to the bonding of Zn with Mg/Na and the expansion of lattice constant. Comparatively, Cu K-edge XANES (Supplementary Fig. 14c) shows a negligible change in the Cu oxidation state at the pristine and fully charged state, but Cu EXAFS (Supplementary Fig. 14d) shows a shift of Cu-Cu peak to a higher radial distance at the charged state. These all confirm the Na and Mg cations (or metals) absorption into the alloy structure^45^.

### Electrochemical analysis

**Fig. 2:**
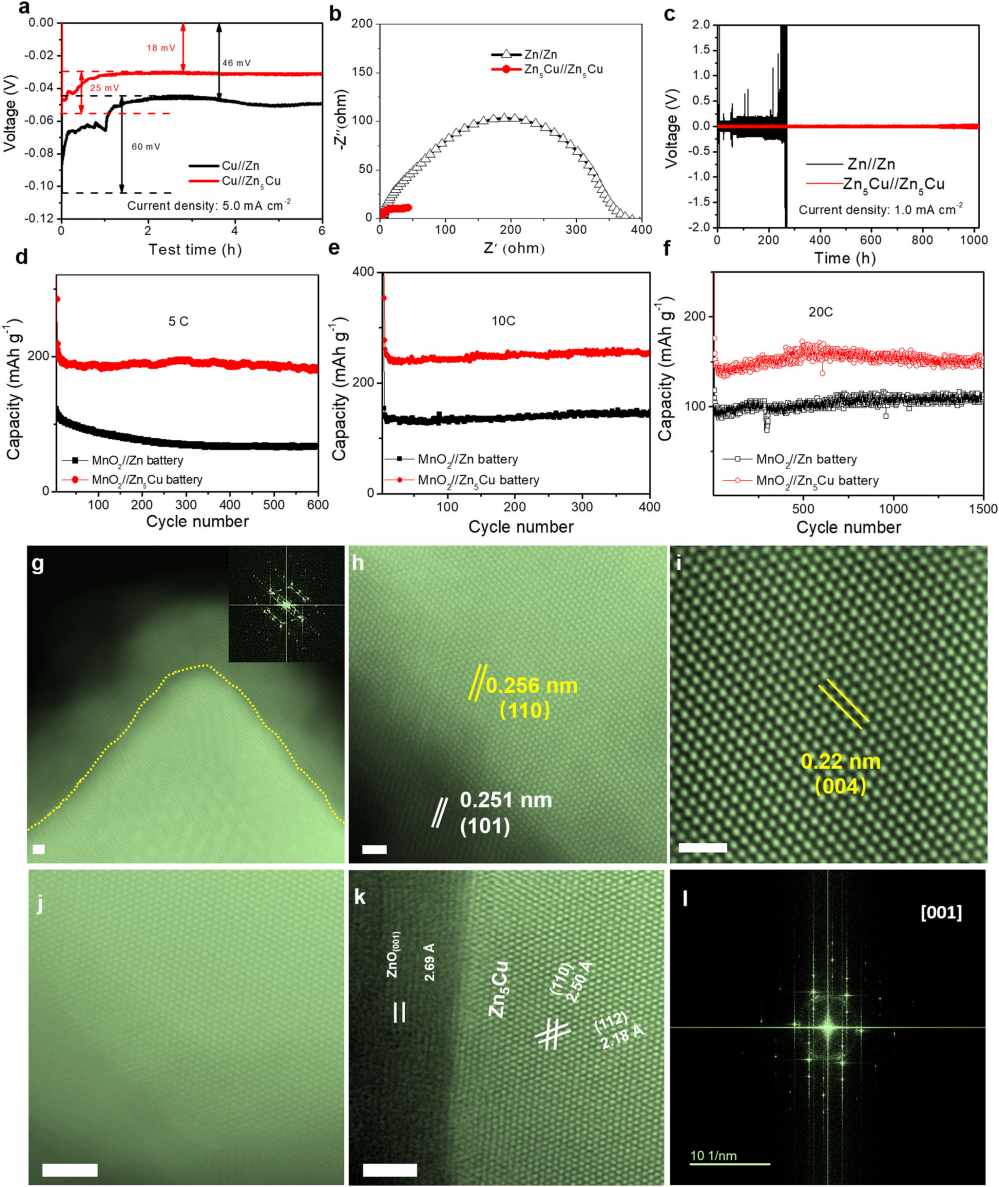
Electrochemical performance and structure characterizations of Zn-Cu anode in aqueous Zn batteries. **a** Nucleation overpotentials of Zn-Cu and Zn asymmetric cells (*vs*. Cu electrode) at a current density of 5.0 mA cm^−2^. **b** Electrochemical impedance spectroscopy (EIS) of Zn//Zn and Zn-Cu//Zn-Cu cells. **c** Long-term stability of Zn//Zn and Zn-Cu//Zn-Cu cells at a current density of 1.0 mA cm^−2^. Long-term stability of Zn-Cu//MnO_2_ batteries in (**d**) dual-cation (Mg^2+^+Zn^2+^) electrolyte at 5 C and in (**e**, **f**) dual-cation (Na^+^+Zn^2+^) electrolyte at 10 C and 20 C, respectively. STEM images of (**g**–**i**) Mg-Zn_5_Cu (Scale bar: 1.0 nm) and (**j**, **k**) Na-Zn_5_Cu (Scale bar: 2.0 nm) after 100 cycles.

The reversibility and long-term stability of the Zn-Cu anode in the aqueous Zn batteries were investigated in both symmetric and asymmetric cells. Firstly, the nucleation and plateau overpotentials, which present the formation and growth thermodynamics of the Zn nucleus in the initial plating process^36^, were tested as shown in Fig. 2a. The lower nucleation overpotential suggests more nucleation sites for Zn plating leading to uniform Zn deposition^37^. Compared with the pristine Zn (60 mV and 46 mV), the Zn-Cu anode has lower nucleation and plateau overpotentials (25 mV and 18 mV) under a current density of 5.0 mA cm^−2^. Electrochemical impedance spectra (EIS) of symmetric Zn-Cu//Zn-Cu cells were employed to evaluate the charge-transfer kinetics compared with symmetric Zn//Zn cells. In a single-cation electrolyte (2 M ZnSO_4_), a remarkably reduced charge-transfer resistance of 43 ohms was achieved in the Zn-Cu//Zn-Cu cell (Fig. 2b), which was 8 times lower than that of the Zn//Zn cell (382 ohms), indicating a greatly improved charge-transfer kinetics. After cycling, the charge-transfer resistance of the Zn-Cu//Zn-Cu cell was well-maintained at lower values than that of the Zn//Zn cell, double-confirming the excellent dynamics at the solid/liquid interface (Supplementary Fig. 15). We further tested the cyclic voltammetry (CV, Supplementary Fig. 16) curves of the single-cation Zn electrolyte (2 M ZnSO_4_) in the three-electrode system using the Zn-Cu alloy as reference and counter electrodes, showing high reversibility even after 50 cycles. The cycling stability of the Zn-Cu anode was evaluated by galvanostatic cycling in the symmetric Zn-Cu//Zn-Cu cell, which shows ultra-stable Zn plating/stripping behaviors for over 1000 h at 1.0 mA cm^−2^. In contrast, the symmetric Zn//Zn cell experienced short-circuit after only 230 h (Fig. 2c and Supplementary Fig. 17). The electrochemical performance of the Zn-Cu and Zn anode at a low current density (< 1.0 mA cm^−2^) during the Zn deposition/stripping processes have also been conducted. Under low current densities of 0.3 mA cm^−2^ and 0.5 mA cm^−2^, the 3D structured Zn-Cu anode shows a superior electrochemical performance (Supplementary Fig. 18). The long-term galvanostatic cycling performance of symmetric Zn-Cu and pristine Zn cells at a current density of 10 mA cm^−2^ (areal capacity: 1 mAh cm^−2^) for over 300 h confirmed that the Zn-Cu anode has a superior electrochemical performance even under higher areal capacity and higher current densities after long-term cycling compared with pristine Zn metal anode (Supplementary Fig. 19). The cycling performance of symmetric Zn-Cu and pristine Zn cells at a current density of 20 mA cm^−2^ (areal capacity: 2 mAh cm^−2^) at 45 °C for over 160 h confirms that the Zn-Cu anode has a superior electrochemical performance even at a higher temperature (45 °C) compared with pristine Zn metal anode (Supplementary Fig. 20). We also tested the electrochemical performance of the Zn-Cu alloy with different content of Cu, as shown in Supplementary Fig. 21. The symmetric Zn-Cu//Zn-Cu cells show that the Zn-Cu alloy with a higher Cu content has a lower overpotential and also exhibits an excellent cycling performance even after long-term (900 h) Zn plating/stripping processes. Different current densities from 0.5 mA cm^−2^ to 50 mA cm^−2^ were employed to analyze the Zn plating processes by ex-situ SEM (Supplementary Fig. 22). A smooth surface of the Zn-Cu anode without dendrite growth was observed under different current densities, even at high a current density of 50 mA cm^−2^, exhibiting promising potential for dendrite-free aqueous batteries. When tested in the Zn-Cu//Cu half-cell, the average Coulombic efficiency (CE) of the Zn-Cu anode increases from 93.3% in the initial cycle to 99.5% after 200 cycles (Supplementary Fig. 23) at a current density of 5 mA cm^−2^ (areal capacity: 0.5 mAh cm^−2^). By contrast, the Zn//Cu half-cell shows a lower average CE of 99.0% after 110 cycles with an initial CE of 86.6%. Supplementary Fig. 24a shows the Zn plating/stripping profiles under different current densities from 0.1 to 10 mA cm^−2^. The overpotential of the Zn-Cu//Zn-Cu cell is lower than that of the Zn//Zn cell under different current densities, indicating the excellent rate performance of the Zn-Cu anode. The Zn plating/stripping profiles tested at 5.0 mA cm^−2^ (Supplementary Fig. 24b, c) show greatly reduced overpotentials of Cu//Zn-Cu cell (105 mV) compared with that of Cu//Zn cell (256 mV) in the initial cycle. After 10 cycles of activation, the CE of the Cu//Zn-Cu cell is much higher than that of the Cu//Zn cell, confirming the excellent reversibility of Zn plating/stripping processes on the Zn-Cu anode.

The Zn plating/stripping processes on the pristine Zn anode in aqueous Zn batteries have been widely studied in recent years. However, few reports are investigating the effect of dual-cation electrolytes on the alloyed anode and cathode materials of aqueous Zn batteries. We assembled coin cells using MnO_2_ cathode, Zn-Cu (Zn_5_Cu) anode, and hybrid electrolytes (Electrolyte 1: 1 M ZnSO_4_ + 1 M Na_2_SO_4_; Electrolyte 2: 1 M ZnSO_4_ + 1 M MgSO_4_). As shown in Fig. 2d, the MnO_2_//Zn_5_Cu cell in the Mg^2+^-containing hybrid “Electrolyte 2” shows a higher capacity of 183.2 mAh g^−1^ than that of the MnO_2_//Zn cell (67.6 mAh g^−1^) after 600 cycles at 5 C. Compared with the MnO_2_//Zn_5_Cu cell in the dual-cation “Electrolyte 2”, the MnO_2_//Zn_5_Cu cell in a single-cation electrolyte shows a relatively stable cycling performance but exhibits a lower capacity compared with the MnO_2_//Zn_5_Cu cell in the dual-cation electrolyte (Supplementary Fig. 25). The results confirm that the synergetic effect of the 3D interface material and dual-cation electrolytes would favor the excellent electrochemical performance of the aqueous battery systems. We tested the self-discharge of the Zn-Cu//MnO_2_ battery at 45 °C. The open-circuit voltage (OCV) of the Zn-Cu//MnO_2_ cell was kept very stable even after over 70 h without any degradation (Supplementary Fig. 26), confirming the stability of the Zn-Cu//MnO_2_ battery in the Mg^2+^-containing dual-cation electrolyte. The improved charge-transfer kinetics (Supplementary Fig. 27) can enhance the dynamics of cells in ion storage processes, thus could increase the capacity for dual-cation battery systems using Na-containing electrolytes at a high rate. Furthermore, at a higher rate of 10 C (Fig. 2e), the MnO_2_//Zn_5_Cu cell in Na^+^-containing hybrid “Electrolyte 1” delivers a higher capacity of 262.6 mAh cm^−2^ for the MnO_2_//Zn_5_Cu cell. Contrastly, the MnO_2_//Zn cell only exhibits a capacity of 148.6 mAh g^−1^ after 400 cycles. Even at 20 C (Fig. 2f), the MnO_2_//Zn_5_Cu cell in the Na^+^-containing hybrid electrolyte keeps very stable performance over 1500 cycles with a high capacity of 149.1 mAh g^−1^, which is among the most competitive aqueous Zn batteries (Supplementary Table 1).

The charge-transfer kinetics of the Zn-Cu//Zn-Cu cell investigated by EIS (Supplementary Fig. 28) demonstrate a remarkably reduced charge-transfer resistance than the Zn//Zn cell in dual-cation electrolytes, confirming the excellent dynamics of the Zn-Cu anode. The enhanced charge-transfer kinetics for aqueous batteries will facilitate the rate performance^46^. The CV curves of the Zn-Cu anode tested in the dual-cation electrolytes (Supplementary Fig. 29) illustrate highly reversible Zn plating/stripping behaviors in a voltage range of −0.6 to 2 V using Zn-Cu alloy as reference and counter electrodes. Moreover, the Zn-Cu alloy shows a more stable performance without any fluctuation in CV curves compared with the pristine Zn in the dual-cation electrolytes. Meanwhile, the Mg^2+^-containing hybrid electrolyte (Electrolyte 2: 1 M ZnSO_4_ + 1 M MgSO_4_) has broadened the voltage window up to 2.6 V (Supplementary Fig. 30). The Na^+^-containing hybrid electrolyte (Electrolyte 2: 1 M ZnSO_4_ + 1 M Na_2_SO_4_) has a similar voltage window (up to ~2.5 V), confirming the good stability and promising application prospect of Zn-Cu anode in the dual-cation electrolytes. With the improved electrode/electrolyte interface compatibility, anti-corrosive abilities, and enhanced charge-transfer kinetics for 3D Zn-Cu anode, the MnO_2_//Zn_5_Cu cells exhibit higher capacities and excellent rate performance in dual-cation electrolytes.

### 3D Alloy characterizations in dual-cation electrolytes

The MnO_2_//Zn-Cu cell was disassembled after cycling in the hybrid electrolytes. The cycled Zn-Cu anode was washed several times to fully eliminate the residual salts. We have conducted X-ray photoelectron spectroscopy (XPS) characterizations on the fresh and cycled Zn-Cu anodes in the dual-cation electrolyte. Within our expectation, we could observe the as-prepared sample with Cu (931.3 eV for Cu 2p3/2 and 951.3 eV for Cu 2p1/2) and Zn (1021.3 eV for Zn 2p3/2 and 1044.3 eV for Zn 2p1/2) in both metallic states from XPS, which confirms the formation of Zn-Cu alloy^31^, as shown in the Supplementary Fig. 31. After cycling, as shown in Supplementary Fig. 32, we can also observe the ZnO (1020.4 eV for 2p3/2 and 1043.6 eV for 2p1/2)^47^, the coexistence of the metallic state (1022.2 eV for 2p3/2 and 1045.3 eV for 2p1/2), and oxidation state of Zn reveals a thin ZnO formed on the surface of the 3D electrode^48^. The ZnO coating layer plays a positive role in stabilizing the long-term cycling performance by suppressing the dendrite growth, lowering the overpotential of the nucleation barrier, and reducing the side reactions, which could efficiently guide the ion transport during the uniform stripping and deposition process^36,49^. After cycling in the Mg^2+^-containing hybrid electrolyte (Electrolyte 2: 1 M ZnSO_4_ + 1 M MgSO_4_), the surface ZnO layer remains an epitaxial interface with the Zn_5_Cu matrix, which is consistent with the as-prepared sample (Fig. 2g). However, the lattice spacings of Zn_5_Cu (110) and (004) were expanded to 2.56 Å and 2.21 Å in comparison with the original 2.43 Å and 2.13 Å, respectively. Besides, the ZnO (101) also expanded to 2.51 Å (Fig. 2h, i and Supplementary Fig. 33). The EDS mapping (Supplementary Fig. 34) reveals an evenly distributed Mg element in the cycled Zn_5_Cu sample obtained after cycling in an Mg-containing hybrid electrolyte (denoted as Mg-Zn_5_Cu). Consequently, the slightly resident Mg ion inserted in the lattice of the ZnO and Zn_5_Cu matrix attributes to the lattice expansion. On the other hand, cycled Zn_5_Cu sample in Na^+^-containing hybrid electrolyte (denoted as Na-Zn_5_Cu), a thin ZnO layer has also been found to cover on the Zn-Cu electrode (Supplementary Fig. 35)^50^, and the lattice spacings of Zn_5_Cu (110) and (004) were expanded to 2.50 Å and 2.18 Å, respectively (Fig. 2j–l). While the ZnO (001) expanded to 2.69 Å and the Na element was evenly distributed in the forest-like 3D Zn-Cu structure (Supplementary Fig. 36).

### In-situ optical imaging and analysis

**Fig. 3:**
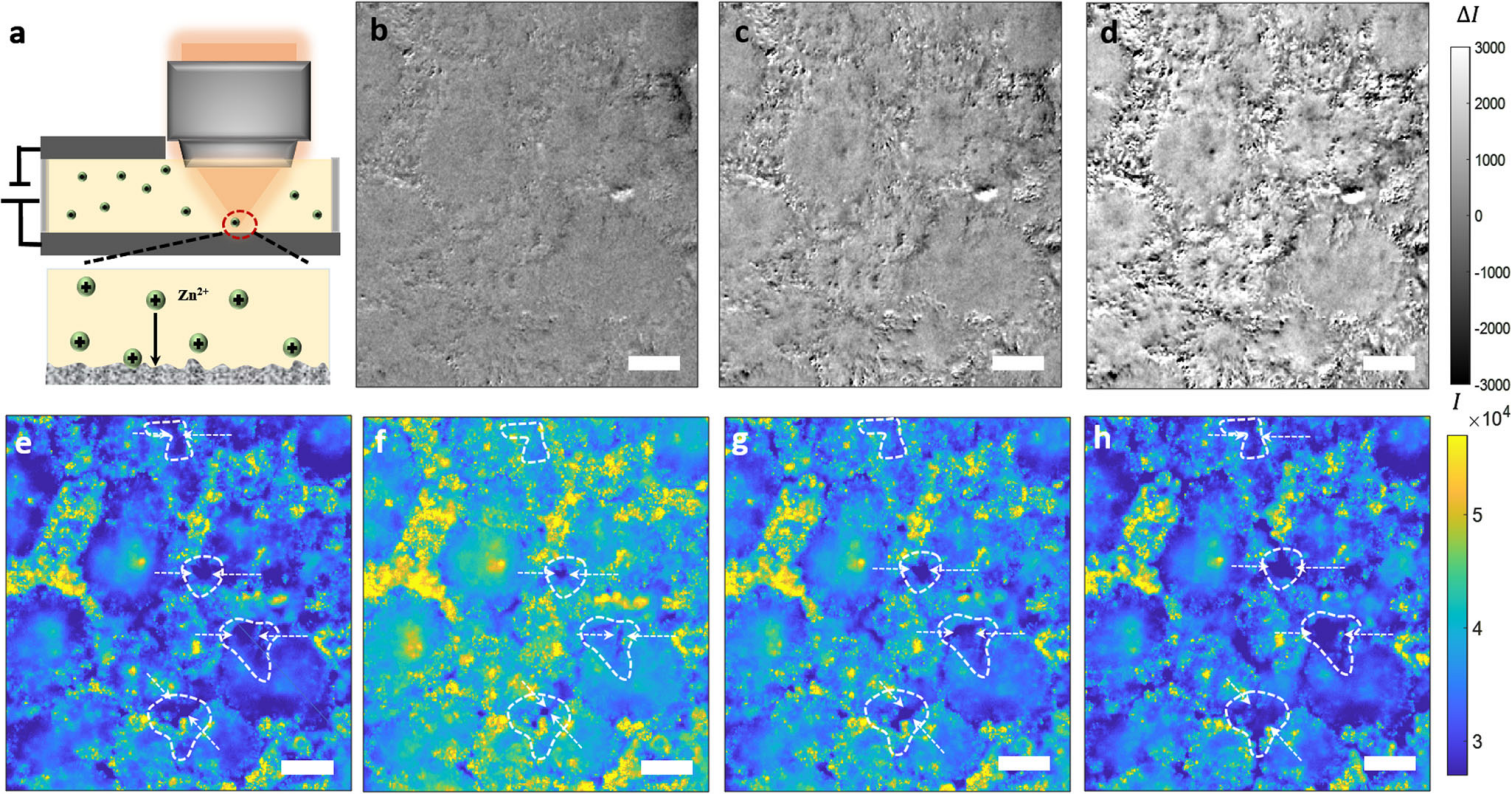
In-situ visualization of Zn plating/stripping on Zn-Cu anode. **a** Experimental setup. **b**–**d** The differential images of Zn-Cu anode in the early stages of Zn plating. The images were taken at 5 s, 10 s, and 20 s of Zn plating, and then subtracted from the first image. The contrast shows the intensity change ($\triangle I$) during the deposition. **e**, **f** Images of the forest-like 3D Zn-Cu anode at 500 s and 1000 s during Zn plating. **g**–**h** Images of the forest-like 3D Zn-Cu anode at 500 s and 1000 s during Zn stripping. Current density: 50 mA cm^−2^; Electrolyte: 1 M ZnSO_4_ + 1 M MgSO_4_; Scale bar: 10 µm.

We designed an aqueous battery cell (Fig. 3a) for in-situ optical imaging of dynamic Zn plating/stripping processes on the 3D nanostructured Zn-Cu anode (Fig. 3b–d) under conditions analogous to the electrochemical environments of Zn batteries. Figure 3e–h shows the snapshots taken during the Zn plating/striping processes on the forest-like Zn-Cu anode. The optical intensity in the images represents the reflection of the electrode surface. The pristine Zn anode was studied first for comparison and understanding of dendrite suppression of the Zn-Cu anode. Supplementary Movie S1 shows very fast nucleation and growth of Zn throughout the entire process on the pristine Zn, forming obvious branch-like dendrites. In addition to the movie shown in Supplementary Movie S1, which demonstrates the obvious dendrite-like Zn plating, we further analyze the details of the morphology evolution of the pristine Zn anode. The initial morphology of the electrode is subtracted to focus on the change caused by Zn deposition. Supplementary Fig. 37a indicates that the very initial deposition is relatively uniform, while as deposition capacity increased, the non-uniform deposition happened quickly, leading to obvious intensity difference across the entire electrode surface as shown in Supplementary Fig. 37b, c. Even though the applied current density is much smaller and the deposition time is much shorter when compared with the Zn-Cu anode. This phenomenon can also be easily observed in the electrode morphology. Supplementary Fig. 37d–f shows the snapshots of the electrode morphologies taken during the Zn plating process. Clearly, the plating happened unevenly, and some regions have obviously more plating happened (marked by the white dash line in Supplementary Fig. 37e, f), while other regions remain almost the same. This non-uniform deposition could further induce the formation of metal dendrites and lead to battery failure. The significant difference when the plating happened on the pristine Zn anode proves the capability of the proposed Zn-Cu anode to regulate uniform Zn plating and the potential for dendrite suppression.

To comprehensively understand the dendrite suppression mechanism of the Zn-Cu anode, the early stages of Zn plating on the forest-like 3D Zn-Cu anode were examined using the in-situ optical microscope. To visualize the initial Zn plating process, we subtracted the first frame from the movie and only observe the optical difference caused by the nucleation and growth of Zn. The Zn plating onto the electrode increases the reflectivity and then increases the intensity of the electrode. Figure 3b–d and Supplementary Movie S2 show the snapshots of the electrode surfaces in the early stages of Zn plating. The intensity increases across the entire electrode surface, indicating the preferential deposition of Zn into the 3D forest-like structure of the Zn-Cu anode. Noticeably, not many bright spots, corresponding to the localized nucleation and growth of Zn, were observed, indicating that the Zn plating in the early stages is fairly uniform. This demonstrates that the Zn-Cu anode can minimize the inhomogeneous deposition of Zn even in dual-cation electrolytes.

The morphology evolution of the forest-like Zn-Cu anode during the Zn plating/stripping processes was continuously monitored as shown in Fig. 3e–h. Comparing the late stage of Zn plating (Fig. 3f) with the initial stage (Fig. 3e), the intensity increases due to the changed surface reflectivity of the electrode. This observation is consistent with Fig. 3b–d. Moreover, the main structures of the electrode do not show any significant morphology changes, indicating that the Zn plating preferentially happens inside the 3D structure rather than on the surface of the Zn-Cu anode. This is because the unique forest-like 3D structure, together with the alloyed surface composition (Zn-Cu), guides the Zn plating process by controlling the location of Zn nucleation, and therefore suppressing the dendritic growth of Zn. To further verify this conclusion, we carefully analyzed the movies and images and compared the representative regions of the electrode as circled in white dashed lines (Fig. 3e–h). During Zn plating, the preferential deposition of Zn inside the 3D structure of Zn-Cu will cause structural expansion. As a result, the voids (white dash circles in Fig. 3e, f) shrunk due to the deposition of Zn inside the structure. During Zn stripping, the voids (white dash circles in Fig. 3g, h) increase because of the removal of Zn from the structure. This phenomenon is shown much clearer in the dynamic movies (Supplementary Movies S3, S4) than in the static images. The in-situ dynamic visualizations demonstrate that the forest-like 3D Zn-Cu anode regulates the Zn plating processes by allowing the preferential deposition of Zn inside the 3D structure. Such Zn growth mode will minimize the possibility of dendrite formation on the Zn-Cu surface. We have also studied the Zn plating/stripping processes at the current density of 5 mA cm^−2^ and 50 mA cm^−2^ on the Zn-Cu anode using the hybrid electrolyte (Supplementary Movies S5–S8). The results show a similar trend that the voids structures on the electrode will expand and shrink during the Zn plating/stripping processes, respectively. When Zn plating happened, the overall electrode structures did not show obvious vertical growth, the alteration mainly happened horizontally causing the structure expansion and gaps to shrink. During the stripping process, reversible structures shrink and gaps between structures’ expansion were observed. Both the experimental results obtained under large current density and small current density help us to understand and explain the dendrite minimization performance of the 3D Zn-Cu anode. The results obtained from the in-situ optical microscope provide future guidance to design a 3D anode to minimize the Zn dendrite formation.

### Ex-situ physicochemical characterizations of electrodes

**Fig. 4:**
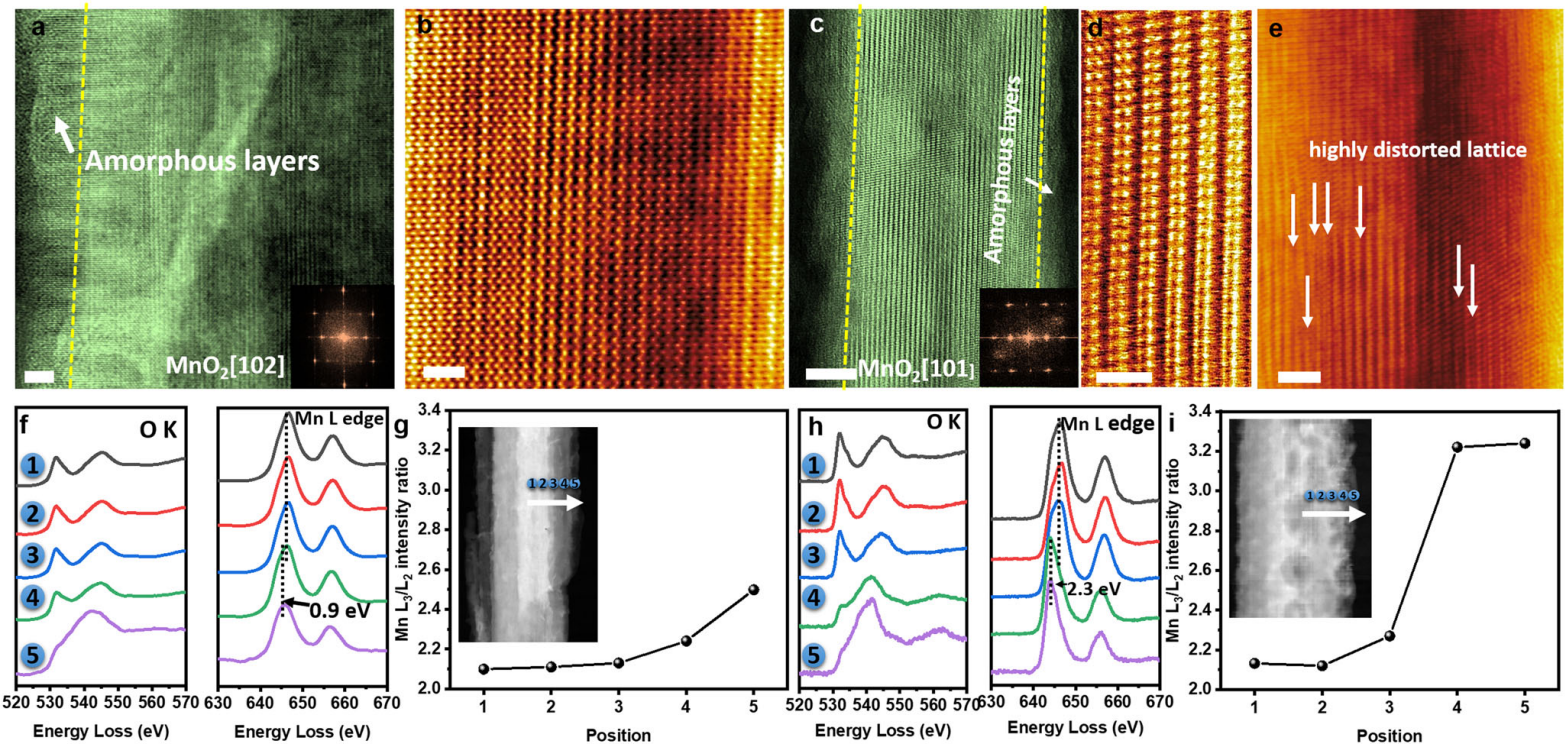
Atomic STEM and O-K edge spectra of Zn batteries in dual-cation electrolytes after 100 charge/discharge cycles at the fully discharged state. **a**, **b** STEM images of Mg-MnO_2_ at the fully discharged state. The inset shows a typical FFT of MnO_2_ [102]. Scale bar: **a** 2.0 nm, **b** 1.0 nm. **c**–**e** STEM images of Na-MnO_2_ at the fully discharged state. The inset shows a typical FFT of MnO_2_ [101]. Scale bar: **c** 5.0 nm, **d** 1.0 nm, **e** 2.0 nm. **f** O-K edge spectra and the Mn-L_2, 3_ white lines for locations “1” to “5” (shown in (**g**)) from core to shell. **g** Mn L_3_/L_2_ intensity ratio as a function of Mn positions (“1” to “5” shown in inset). **h** O-K edge spectra and the Mn-L_2_,_3_ white lines for locations “1” to “5” (shown in (**i**)) from core to shell. **i** Mn L_3_/L_2_ intensity ratio as a function of Mn positions (“1” to “5” shown in inset).

The structure and composition of the MnO_2_ cathode after dual-cation intercalation were examined. After full discharging of MnO_2_//Zn-Cu cell in the Mg^2+^-containing hybrid electrolyte (Electrolyte 2), the crystalline MnO_2_ (denoted as Mg-MnO_2_) was surrounded by an amorphous shell (Fig. 4a, b). EDS mapping reveals that the amorphous layer is composed of Mn and O with 0.23 at.% of Mg incorporation (Supplementary Fig. 38). The atomic structure along MnO_2_ [102] in the core region is shown in Fig. 4b and FFT in Fig. 4a verifies that the core structure remains MnO_2_. While, after full discharging of MnO_2_//Zn-Cu cell in Na^+^-containing hybrid electrolyte (Electrolyte 1), the cycled MnO_2_ (denoted as Na-MnO_2_) also displays an amorphous layer (Fig. 4c) with evenly distributed Na element around MnO_2_ (Supplementary Fig. 39). The core lattice of MnO_2_ coincided well with MnO_2_ [101] zone, indicating core structure preserves well with a pristine structure. However, a high density of dislocations was found in the core of MnO_2_ (Fig. 4d, e) induced by the incorporation of Na^+^. The electronic structure of MnO_2_ after discharging in Mg^2+^-containing (Electrolyte 2) and Na^+^-containing hybrid electrolytes (Electrolyte 1) were explored by investigating the O-*K* and Mn-*L*_2,3_ energy-loss near-edge structure (ELNES) from the core to the shell. The measured positions are marked in the insets of Fig. 4g, i. For the Mg-MnO_2_ cathode, the intensity of O-*K* pre-peaks becomes weaker from the core to the edge, suggesting a gradual valence reduction of Mn due to an increase in oxygen deficiency near the edge (Fig. 4f, g)^51^. Figure 4g shows the intensity ratios of the *L*_3_ and *L*_2_ peaks of the Mn-*L*_2,3_ ELNES in Fig. 4f. The Mn(*L*_3_) main peak of Mn‐*L*_2,3_ shifts from 646.5 eV to 645.6 eV from core to edge, indicating the valence state reduction of Mn. Typically, the white-line ratio for Mn possesses an inverse trend to valence states^52^. The intensity ratios increase from ~2.11 to ~2.5, further confirming the mean Mn valence state gradually decreases near the edge of MnO_2_. The reduced Mn valence state was caused by the formation of the MnOOH layer^53–55^. Also, the XRD pattern of the MnO_2_ cathode after full discharge was characterized as shown in Supplementary Fig. 40. And the merging characteristic peaks matched well with MnOOH (JCPDS 18–0805) and ZnSO_4_[Zn(OH)_2_]_3_·4H_2_O for cycled MnO_2_ cathode at a fully discharged state. Meanwhile, the O-*K* edge ELNES from the core to shell present a similar trend with Mg-MnO_2_, as shown in Fig. 4h. The intensity of O-*K* pre-peaks becomes weaker from the core to the edge, suggesting a gradual valence reduction of Mn. As for Na-MnO_2_, the Mn (*L*_3_) main peak of Mn‐*L*_2,3_ shifted from 646.5 eV to 644.2 eV from core to edge, revealing a lower Mn valence state than Mg-MnO_2_ (Fig. 4h). Besides, the intensity ratios progressively increase from ~2.12 to ~3.23 (Fig. 4i), verifying the mean Mn valence state rapidly decreases near the edge of MnO_2_. In the CV curves (Supplementary Fig. 41), compared with the MnO_2_//Zn battery using the 2 M ZnSO_4_ electrolyte and MnO_2_//Zn_5_Cu battery using the 2 M ZnSO_4_ electrolyte, the MnO_2_//Zn_5_Cu battery using dual-cation electrolytes have a higher discharging plateau, and also have obvious reduction peaks, which could due to the influence of co-intercalation during discharging process in the Mg^2+^-containing electrolyte and the Na^+^-containing electrolyte. The XANES region (Supplementary Fig. 42a) reveals that the bulk MnO_2_ structure is preserved after discharging indicated by the close similarity in line shape and position between the two samples. Combined with the results of CV curves (Supplementary Fig. 41), a comparison of MnO_2_ cathode EXAFS (Supplementary Fig. 42b) at pristine and a fully discharged state shows some slight bond distance changes, which could be due to the intercalation of dual cations. Therefore, in the MnO_2_//Zn-Cu cells using Na^+^-containing and Mg^2+^-containing hybrid electrolytes, the secondary Na^+^ and Mg^2+^ ions can insert the MnO_2_ cathode during discharging. To evaluate the electrochemical behaviors of Mg^2+^, Na^+^, and Zn^2+^ ions after the full charge process, the STEM-mappings of MnO_2_ cathode after 100 charge/discharge cycles at the fully charged state were carried out. Supplementary Figs. 43, S44 indicates the little amount of Mg cations and Na cations in the lattice, from which we can see that the inserted Mg^2+^ and Na^+^ ions are mostly extracted from the MnO_2_ cathode at the fully charged state. In contrast, some Zn ions still remain in the MnO_2_ cathode at charged state in a single-cation electrolyte (2 M ZnSO_4_) as shown by the EDS map in Supplementary Fig. 45. These unique interface materials consisting of 3D alloy structures with well-regulated dual-cations kinetics provide us with an insight into the design of high-performance dual-cation Zn batteries and beyond.

## Discussion

In conclusion, we report a unique interface material composed of 3D structured Zn-Cu alloy for dendrite-free aqueous Zn batteries. The 3D Zn-Cu alloy nanostructures are demonstrated to be in favor of effectively regulating the nucleation and growth kinetics of Zn inside the forest-like 3D nanostructures during Zn plating. An in-situ protocol was designed to observe the plating/stripping processes of the Zn-Cu anode in conditions analogous to aqueous Zn batteries using dual-cation electrolytes at low current densities and high current densities of 30 mA cm^−2^ and 50 mA cm^−2^. The morphology evolutions of the MnO_2_ cathode and Zn-Cu anode in the dual-cation electrolytes were investigated, which provides an insightful understanding of the Zn-Cu anode when used in aqueous Zn batteries. This work paves a new way for designing high-capacity, dendrite-free, and durable anode materials for aqueous batteries and beyond.

## Methods

### Preparation of Zn-Cu anode

Zinc sulfate heptahydrate (ZnSO_4_·7H_2_O, Fisher Chemical), 0.5 M copper(II) sulfate pentahydrate (Fisher Chemical), and 10.8 g boric acid (Powder/Certified ACS, Fisher Chemical) were dissolved into the heated DI water with continuous stirring for 30 min until a mixed transparent solution was obtained as plating solution. A two-electrode electrochemical cell was used to electrodeposit Zn-Cu alloy on Zn foil (Zn-Cu anode) with platinum mesh as the counter electrode at 3 V for 0.5~2.5 h. The prepared Zn-Cu anode was noted as a pristine Zn-Cu anode. The ratio of two metal components in Zn-Cu alloy was controlled by tuning the preparation condition of alloy electrodeposition, such as changing the content of Cu-based precursors (CuSO_4_ salts). To obtain the Zn-Bi and Zn-Ni alloy, bismuth(III) nitrate pentahydrate and nickel sulfate hexahydrate has been chosen as the precursors in the alloy electrodeposition processes. The pristine Zn-Cu was chosen as a control sample. In the whole process, continuous bubbles were released at the interface of the zinc foil which was dissolved in the prepared electrolytes. The formation of the thin ZnO layer on the Zn-Cu alloy-based anode could be obtained during the alloy electrodeposition with dissolved oxygen in electrolytes. The process in the presence of oxygen could be summarized as follows:

1 $${H}_{2}O+\frac{1}{2}{O}_{2}+{2e}^{-}\to {2{OH}}^{-}$$

2 $${{Zn}}^{2+}+{2{OH}}^{-}\to {{Zn}({OH})}_{2}$$

3 $${{Zn}({OH})}_{2}\to {ZnO}+{H}_{2}O$$

The overall reaction in the presence of oxygen is

4 $${{Zn}}^{2+}+\frac{1}{2}{O}_{2}+{2e}^{-}\to {ZnO}$$

### Materials characterizations

High-angle annular dark-field scanning transmission electron microscopy (HAADF-STEM), energy dispersive spectroscopy (EDS) mapping, and electron energy-loss spectroscopy (EELS) were carried out at 300 kV on an FEI Themis G2 electron microscope equipped with double correctors (image and probe correctors). For high-angle annular-dark-field (HAADF) images, the convergence angle and collection angle of the detector were 25.1 and 48–200 mrad, respectively. The STEM images were obtained with a convergent semi-angle of 25.1 mrad. The EELS spectra were recorded using a Gatan Quantum ER camera system with an energy spread ΔE of 0.7 eV. X-ray diffraction patterns (XRD) were characterized on a film XRD system (Panalytical X’celerator multi-element detector with Cu Kα radiation source, λ = 1.54056 Å). The morphologies of the materials were characterized by scanning electron microscopy (SEM, ZEISS ultra 55). The contact angles in this work were measured by a KRüSS GmbH Instrument (DSA25, Germany) using the ADVANCE software with a sessile drop model at 25 °C.

### Electrolyte preparations

The aqueous electrolytes were prepared using different Zn, Mg, Na, and Mn-based salts (ZnSO_4_, MgSO_4_, and Na_2_SO_4_) as salts and DI water as a solvent. Electrolyte 1: 1 M ZnSO_4_ + 1 M Na_2_SO_4_; Electrolyte 2: 1 M ZnSO_4_ + 1 M MgSO_4_; Electrolyte 3: 2 M ZnSO_4_.

### Cathode preparations for Zn batteries

The hydrothermal method was used to prepare MnO_2_ powder. Firstly, 1.014 g MnSO_4_·H_2_O was added to 200 ml DI water. After that, 4 ml of 0.5 M H_2_SO_4_ was added under magnetically stirring for 2 h. Then, 50 ml of 0.1 M KMnO_4_ was added slowly to form a homogenous solution, which was transferred to a Teflon-lined PTFE autoclave vessel for the hydrothermal treatment at 120 °C for 12 h. Lastly, the MnO_2_ powder was filtered and washed using DI water and then dried in a vacuum oven at 60 °C overnight. The obtained MnO_2_ powder was mixed with super P carbon and PVDF binder in weight ratios of 7:2:1 in N-methyl pyrrolidinone (NMP) to prepare a slurry, which was carbon cloth (CC) to form the MnO_2_@CC cathode.

### Electrochemical characterizations

For Cu//Zn (Cu//Zn-Cu) cells, the Cu as cathodes, Zn (or Zn-Cu alloy) foils as anodes, and glass fiber membranes as separators were assembled using different aqueous electrolytes to test the Zn plating/stripping processes. Symmetrical cells in this work were assembled and characterized by using symmetrical Zn (or 3D Zn-Cu alloy) foils as both cathode and anode at different current densities (0.1 mA cm^−2^~50 mA cm^−2^). For a full aqueous Zn-ion battery, the MnO_2_@CC cathodes, Zn foils (or 3D Zn-Cu alloy) anodes, and glass fiber membranes were assembled in CR2032 coin cells. The mass loading of MnO_2_ for a full aqueous Zn-ion battery is 3~4 mg cm^−2^ per cell. The amount of electrolytes (100 μl) for each cell is the same. The active areas of electrodes were 1.0 cm^2^ (1 cm×1 cm) in coin cells. Cyclic voltammetry (CV) of the MnO_2_//Zn and MnO_2_//Zn-Cu batteries was measured by CHI 600E electrochemical workstation at a scan rate of 0.1 mV s^−1^. The electrochemical performance of the aqueous electrolytes was tested in a three-electrode setup (Pt mesh as the working electrode, Zn (or Zn-Cu electrode) foil as both counter and reference electrodes) at a scan rate of 1 mV s^−1^. EIS spectra of the MnO_2_//Zn and MnO_2_//Zn-Cu batteries were measured in 2032 coin cells by using an AC amplitude of 10 mV in a frequency range from 100 kHz to 0.01 Hz via a CHI 600E electrochemical workstation

### In-situ visualization

A special aqueous battery cell for in-situ optical imaging of Zn plating/stripping processes was designed with the 3D Zn-Cu as the anode at the bottom and Zn foil as counter and reference electrodes on top of the anode, and polydimethylsiloxane (PDMS) was employed to build up the side walls and hold the electrolyte. During the in-situ visualization process, the objective, the top, and bottom electrodes were all immersed in the electrolyte. To minimize the refractive index mismatch between the air and electrolyte, a 20X water immersion objective (working distance: 2 mm, N.A. 1.0, Thorlab) objective was submerged into the electrolytes and the clear reflected images of the electrode surface could be then obtained. To perform the in-situ visualization, we extended the bottom anode substrate and imaged the morphology changes by the reflected light intensity on the extended area, since the visible light cannot penetrate through the Zn foil electrode. Note that the observation area is not directly facing the top electrode, and the reaction current density could be smaller than the area underneath the top electrode due to the uneven electric field distribution around the edge. To minimize this effect, the observation area was chosen as close as possible to the projection of the top electrode (within 200–400 µm). We have applied COMSOL simulation to calculate the current density distribution along the bottom anode and certified that the current density in the visualization area is about 95% of the area that is directly facing the top electrode, verifying that the observation area can reflect the changes inside the battery electrodes. The samples were imaged in the dual-cation electrolytes under current densities of 5 mA cm^−2^, 30 mA cm^−2^, and 50 mA cm^−2^. The optical focus was carefully adjusted during the measurements to take clear and blurry-free images of the Zn-Cu anode with 3D height variants in the range of 20–30 µm.

### Theoretical calculations

We used the density functional theory (DFT) computations with the Vienna Ab initio Simulation Package (VASP)^56,57^. The Perdew-Burke-Erzenhof (PBE) functional with generalized gradient approximation (GGA) was employed to describe the exchange-correlation energy^58^. A projector-augmented wave (PAW) technique with a cutoff of 520 eV was applied to describe the ion-electron interaction^59^. The total energy convergence tolerance for geometry optimization is less than 1.0 × 10^−6^ eV/atom. The max force is less than 0.01 eV/Å. The van der Waals interaction was considered through the zero-damping corrections DFT-D3^60^. Five-layer Zn(001) facets (3 × 3 supercell), CuZn_3_(001) facets (2 × 2 supercell), CuZn_5_(001) facets (2 × 2 supercell), and Cu_5_Zn_8_(001) facets (1 × 1 supercell) were constructed as the Zinc and Cu-Zn alloy substrates. Four-layer Zn-terminated ZnO (001) facets (4 × 4 supercell) were used for the oxide layer. The surface Brillouin zone was described by a 4 × 4× 1 grid mesh for Zn(001) and ZnO(001), and a 3 × 3 × 1 grid mesh for the Zn-Cu alloy models, respectively. A vacuum region of 15 Å was used to avoid interactions between the slabs along the z-direction. When optimizing the adsorption energies in this work, the top two layers of slab models with the adatom were allowed to relax, remaining the rest layer atoms fixed.

## Supplementary information

Supplementary Information

Description of Additional Supplementary Files

Supplementary Movie 1

Supplementary Movie 2

Supplementary Movie 3

Supplementary Movie 4

Supplementary Movie 5

Supplementary Movie 6

Supplementary Movie 7

Supplementary Movie 8

## Data Availability

The data that support the findings of this study are available within the article and its Supplementary Information file. The source data are available from the corresponding author upon reasonable request.
