# Supplementary material for: Three-dimensional Zn-based alloys for dendrite-free aqueous Zn battery in dual-cation electrolytes
Source: Nat Commun. 2022 Dec 23;13:7922. doi: 10.1038/s41467-022-35618-2 (PMC9789050; doi:10.1038/s41467-022-35618-2)
Supplement: Supplementary file 1 — Supplementary Information [file 41467_2022_35618_MOESM1_ESM.pdf]

Supplementary Information for

Three-dimensional Zn-based Alloys for Dendrite-free Aqueous Zn Battery in Dual-cation Electrolytes

Huajun Tian[1,2 †], Guangxia Feng[3, †], Qi Wang[4, †], Zhao Li[2,5 †], Wei Zhang[2,5], Marcos Lucero[6], Zhenxing Feng[6], Zi-Le Wang[1],Yuning Zhang[1], Cheng Zhen[4], Meng Gu[4,], Xiaonan Shan[3,*], Yang Yang[2,5,7,8,9*]*

[1] Key Laboratory of Power Station Energy Transfer Conversion and System (North China Electric Power University), Ministry of Education, North China Electric Power University, Beijing, 102206 China
[2] NanoScience Technology Center, University of Central Florida, Orlando, Florida 32826, USA
[3] Electrical and Computer Engineering Department, W306, Engineering Building 2, University of Houston, Houston, TX 77204, USA
[4] Department of Materials Science and Engineering, Southern University of Science and Technology, Shenzhen 518055, China
[5] Department of Materials Science and Engineering, University of Central Florida, Orlando, Florida 32826, USA
[6] School of Chemical, Biological, and Environmental Engineering, Oregon State University, Corvallis, OR 97331, USA
[7] Renewable Energy and Chemical Transformation Cluster, University of Central Florida, Orlando, Florida 32826, USA
[8] Department of Chemistry, University of Central Florida, Orlando, Florida 32826, USA
[9] The Stephen W. Hawking Center for Microgravity Research and Education, University of Central Florida, Orlando, Florida 32826, USA

Corresponding authors: Yang.Yang@ucf.edu, xshan@central.uh.edu, gum@sustech.edu.cn

[†] These authors contributed equally to this work

Descriptions for the Supplementary Movie files

Supplementary Movie 1.

Description: Dendrite growth on the pristine Zn surface. The movie was recorded with a 20X water immersion objective and 26 frames per second. The experiment was performed at a constant current density of 30 mA cm^{-2} for 720s.

Supplementary Movie 2.

Description: Differential optical movie. The movie was recorded with a 20X water immersion objective and 5 frames per second under a constant current density of 50 mA cm^{-2} for the 1200s, obtained by subtracting the first frame.

Supplementary Movie 3.

Description: Zn plating process in electrolyte 2. The movie was recorded with a 20X water immersion objective and 5 frames per second under a constant current density of 50 mA cm^{-2} for the 1200s. The plating process in electrolyte 2 was repeated 5 times to demonstrate the morphology change.

Supplementary Movie 4.

Description: Zn stripping process in electrolyte 2. The movie was recorded with a 20X water immersion objective and 5 frames per second under a constant current density of 50 mA cm^{-2} for the 1200s. The stripping process in electrolyte 2 was repeated 5 times to demonstrate the morphology change.

Supplementary Movie 5.

Description: Zn plating process in electrolyte 1. The movie was recorded with a 20X water immersion objective and 5 frames per second under a constant current density of 50 mA cm^{-2} for 600s. The plating process in electrolyte 1 was repeated 5 times to demonstrate the morphology change.

Supplementary Movie 6.

Description: Zn stripping process in electrolyte 1. The movie was recorded with a 20X water immersion objective and 5 frames per second under a constant current density of 50 mA cm^{-2} for 900s. The stripping process in electrolyte 1 was repeated 5 times to demonstrate the morphology change.

Supplementary Movie 7.

Description: Small current density Zn plating process. The plating was performed in a single cation Zn electrolyte (2M $ZnSO_4$) under the current density of 5 mA cm^{-2} for 3000s and the movie was recorded with a 20X water immersion. The entire process was repeated 5 times to better demonstrate the morphology change.

Supplementary Movie 8.

Description: Small current density Zn stripping process. The stripping was performed in 2M $ZnSO_4$ under the current density of 5 mA cm^{-2} for 3000s and the movie was recorded with a 20X water immersion objective. The stripping process was repeated 5 times to exhibit the morphology change.

Supplementary Table S1. Electrochemical performance of the state-of-the-art Zn batteries.

Cathode	Anode	Electrolyte	Rate performance	Cycle performance	Ref.
**MnO_2**	**Zn-Cu**	**1M $ZnSO_4$+1M $MgSO_4$**	**183.2 mAh g^{-1} (at 1.54 A g^{-1})**	**83.7% retention after 600 cycles (at 1.54 A g^{-1})**	**This work**
**MnO_2**	**Zn-Cu**	**1M $ZnSO_4$ +1M Na_2SO_4**	**262.6 mAh g^{-1} (at 3.08 A g^{-1}); 150.8 mAh g^{-1} (at 6.16 A g^{-1})**	**94.8% retention after 435 cycles (at 6.16 A g^{-1}); 85.5% retention after 1500 cycles (at 6.16 A g^{-1})**	**This work**
α-MnO_2 nanorod	Zn	1 M $ZnSO_4$	100 mAh g^{-1} (at 0.63 A g^{-1})	~76% retention after 100 cycles (at 0.63 A g^{-1})	1
MnO_2	Zn	1 M $ZnSO_4$	154 mAh g^{-1} (at 3.0 A g^{-1})	~75.3% retention after 200 cycles (at 3.0 A g^{-1})	2
Od-MnO_2	Zn	1M $ZnSO_4$ +0.2 M $MnSO_4$	~ 120 mAh g^{-1} (at 5.0 A g^{-1})	~84% retention after 2000 cycles (at 5.0 A g^{-1})	3
Mesoporous γ-MnO_2	Zn	1 M $ZnSO_4$	150 mAh g^{-1} (at 0.0005 A cm^{-2})	~60% retention after 40 cycles (at 0.5 A cm^{-2})	4
δ-MnO_2	Zn	1 M $ZnSO_4$	252 mAh g^{-1} (at 0.083 A g^{-1})	~44% after 100 cycle (at 0.083A g^{-1})	5
MnO_2/ PEDOT	Zn	PVA+3M LiCl+2M $ZnCl_2$+0.4M $MnSO_4$	367 mA h g^{-1} (at 0.74 A g^{-1})	83.7% after 300 cycles (at 0.74 A g^{-1})	6
MnO_2	Zn	3 M $Zn(CF_3SO_3)_2$	144 mAh g^{-1}	~94% retention after 2000 cycles (at 2.0 A g^{-1})	7

		+ 0.1 $Mn(CF_3SO_3)_2$	(at 2.0 A g^{-1})		
LiV_3O_8	Zn	1 M $ZnSO_4$	172 mAh g^{-1} (at 0.133 A g^{-1})	~75% retention after 65 cycles (at 0.133 A g^{-1})	8
α-V_2O_5	Zn	21 M bis(trifluoromethane sulfonyl)imide + 1 M $Zn(CF_3SO_3)_2$	156 mAh g^{-1} (at 1.0 A g^{-1})	~80% retention after 2000 cycles (at 1.0 A g^{-1})	9
$Mg_{0.25}V_2O_5$	Zn	3 M $Zn(CF_3SO_3)_2$	90 mAh g^{-1} (at 5.0 A g^{-1})	~97% retention after 2000 cycles (at 1.0 A g^{-1})	10
VS_2	Zn	1 M $ZnSO_4$	138 mAh g^{-1} at 0.5 A g^{-1}	~80% retention after 200 cycles (at 0.5 A g^{-1})	11
$Zn_3[Fe(CN)_6]_2$	Zn	1 M $ZnSO_4$	53 mAh g^{-1} at 0.3 A g^{-1}	~81% retention after 200 cycles (at 0.3 A g^{-1})	12
$ZnMn_2O_4$+C	Zn	3 M $Zn(CF_3SO_3)_2$	72 mAh g^{-1} at 2.0 A g^{-1}	~94% retention after 500 cycles (at 0.5 A g^{-1})	13
$Na_{0.33}V_2O$	Zn	3 M $Zn(CF_3SO_3)_2$	230 mAh g^{-1} at 1.0 A g^{-1}	~93% retention after 1000 cycles (at 1.0 A g^{-1})	14
$Na_2V_6O_{16}$	Zn	1 M $ZnSO_4$	150 mAh g^{-1} at 14.4 A g^{-1}	~80% retention after 1000 cycles (at 14.44 A g^{-1})	15

Supplementary Figures

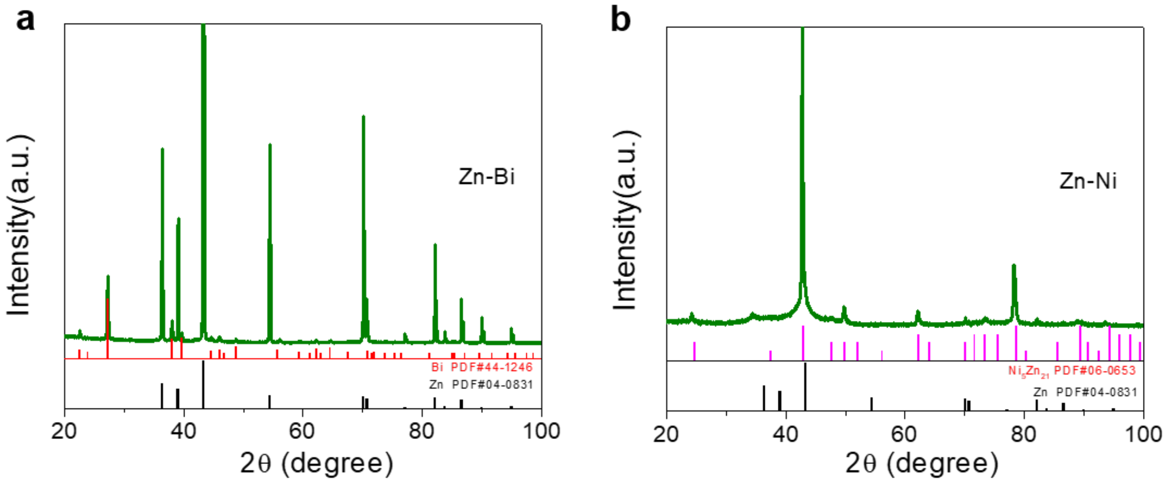

Supplementary Figure 1. XRD patterns of as-prepared Zn-M (M = Bi and Ni) alloy by modifying Bi-containing and Ni-containing precursors and changing prepared conditions via the alloy electrodeposition method.

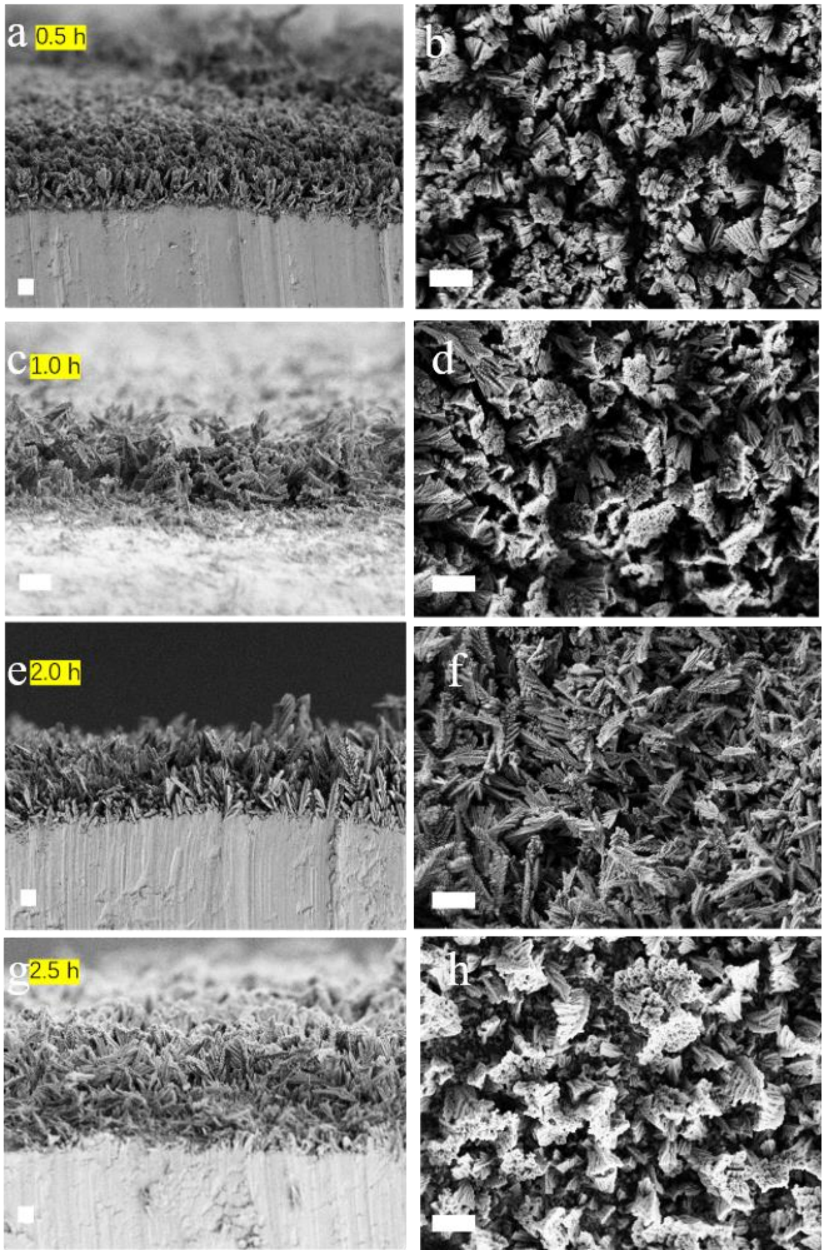

Supplementary Figure 2. Cross-sectional (left column) and surface (right column) SEM images of 3D Zn-Cu anodes. Scale bar: 10.0 μm. A two-electrode electrochemical cell was used to electrodeposit Zn-Cu alloy on Zn foil with platinum mesh as the counter electrode at 3 V. The morphologies of two Zn-Cu alloys under different electrodeposition times can be tuned. It can be seen that the Zn-Cu alloy will become more densely packed after the alloy electrodeposition process for 2.5 h compared with that of 0.5 h.

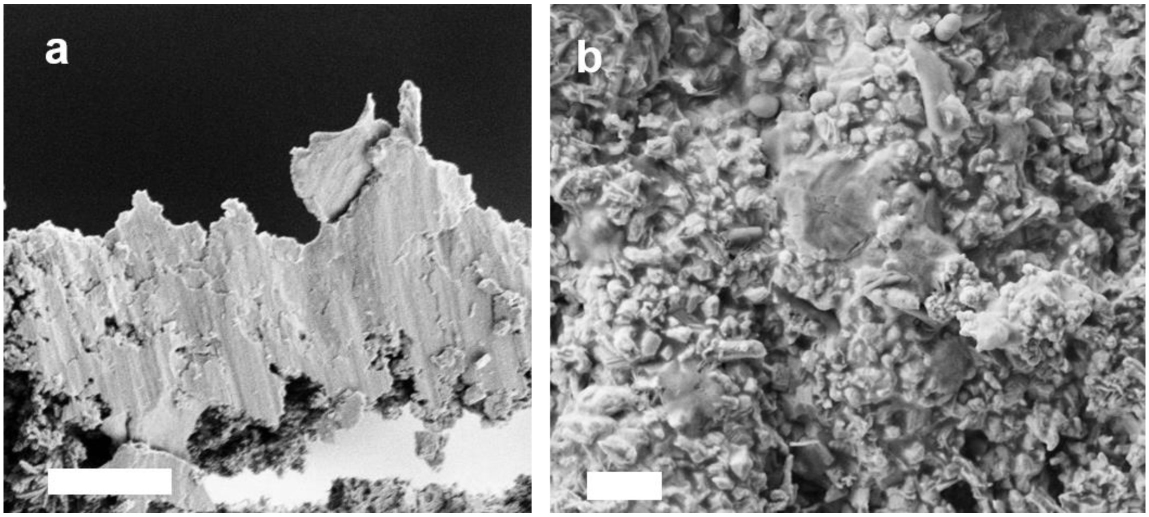

Supplementary Figure 3. (a) Cross-sectional and (b) surface SEM images of flat and dense Zn-Cu anodes. Scale bar: (a) 10.0 μm and (b) 2.0 μm. We used the alloy electrodeposition strategy to prepare the dense-like Zn-Cu alloy anodes, which confirms that this alloy electrodeposition method has a potential advantage to prepare different alloy anodes with different morphologies.

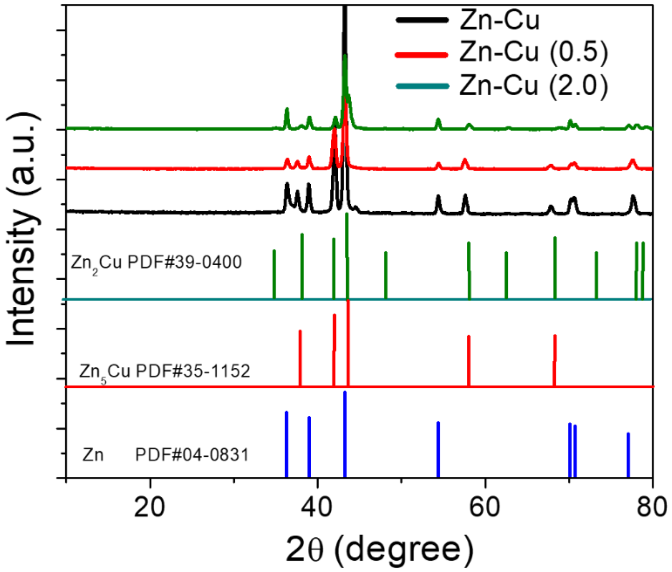

Supplementary Figure 4. XRD patterns of as-prepared Zn-Cu alloy using different-content precursors ($CuSO_4$) in the alloy electrodeposition method. The pristine Zn-Cu was chosen as a reference sample. The XRD patterns confirm that the ratio of two metal components in Zn-Cu alloy can be tuned by changing the content of Cu-based precursors ($CuSO_4$ salts).

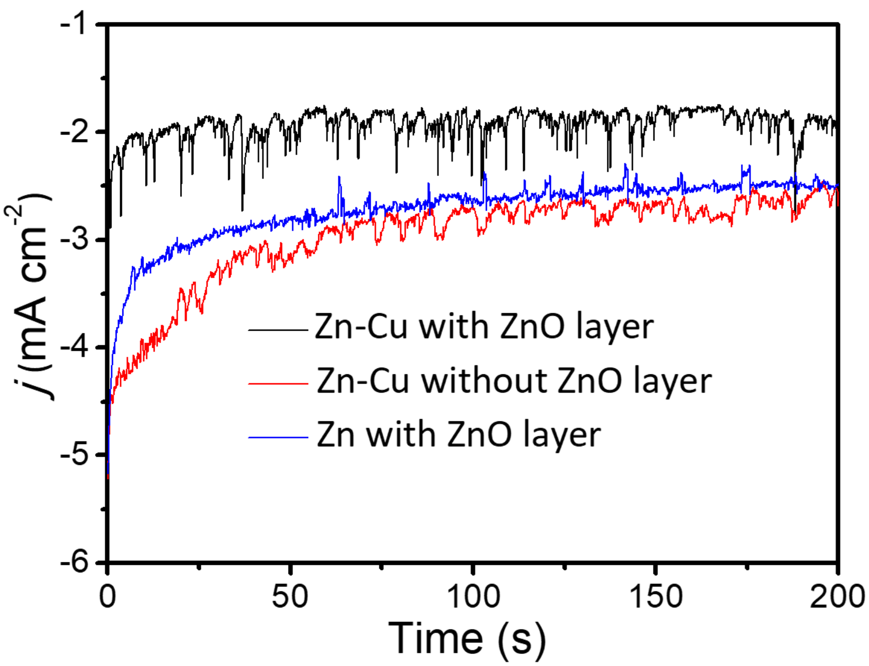

Supplementary Figure 5. I-t test under -0.65 V vs RHE. The *in-situ* gas analysis corresponding to hydrogen evolution for Zn-Cu alloy demonstrates that the current density corresponding to hydrogen evolution for Zn-Cu alloy without a ZnO layer is higher than that of one with a ZnO layer. It confirms that a thin ZnO as a protective layer would be in favor of inhibiting electrode corrosion.

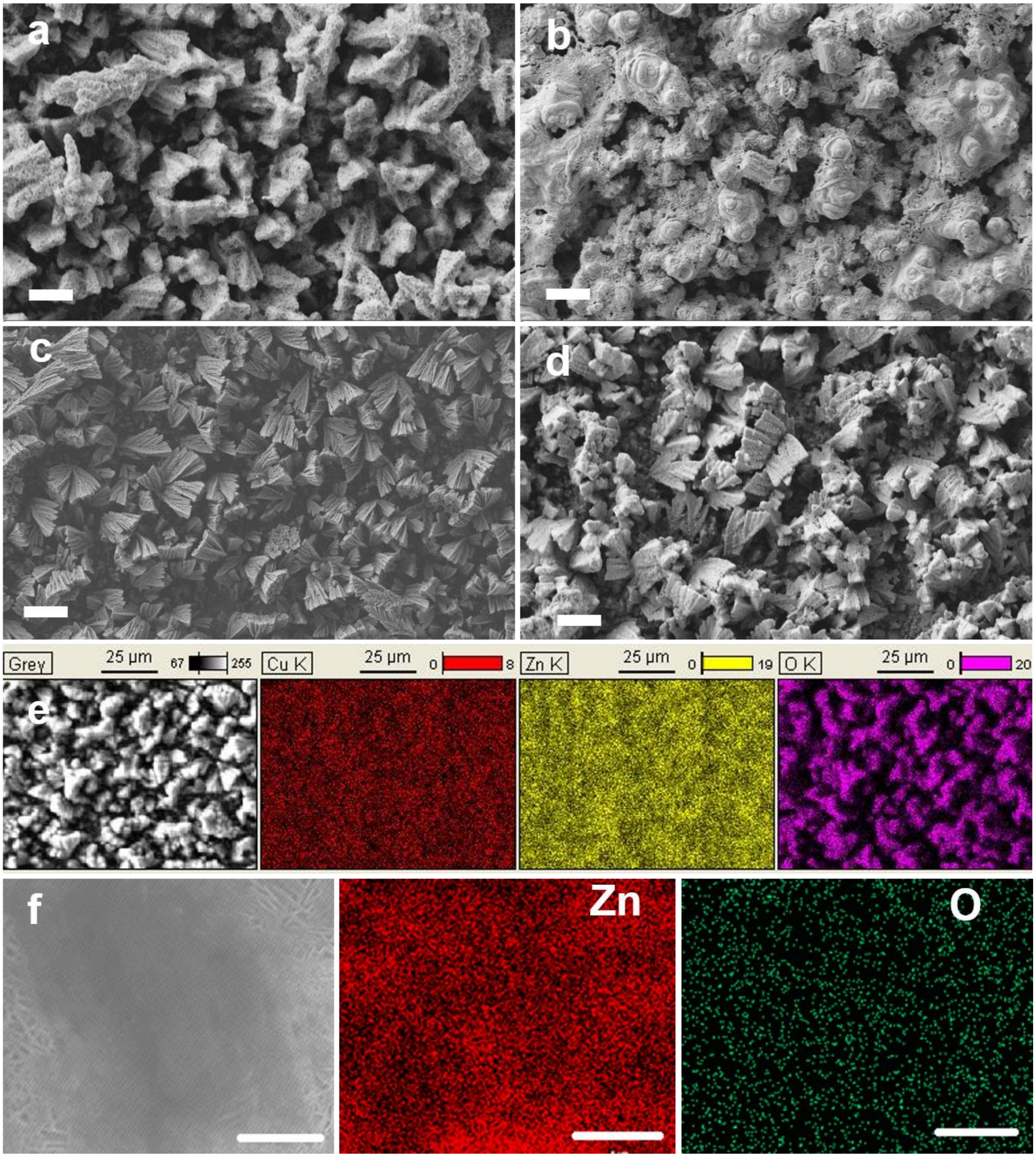

Supplementary Figure 6. SEM images of Zn-Cu anode without ZnO layer (a) before and (b) after stability test. Zn-Cu anode with ZnO layer (c) before and (d) after the stability test, and (e) corresponding EDS mapping after the stability test. (f) SEM image and EDS mapping of pristine Zn anode.

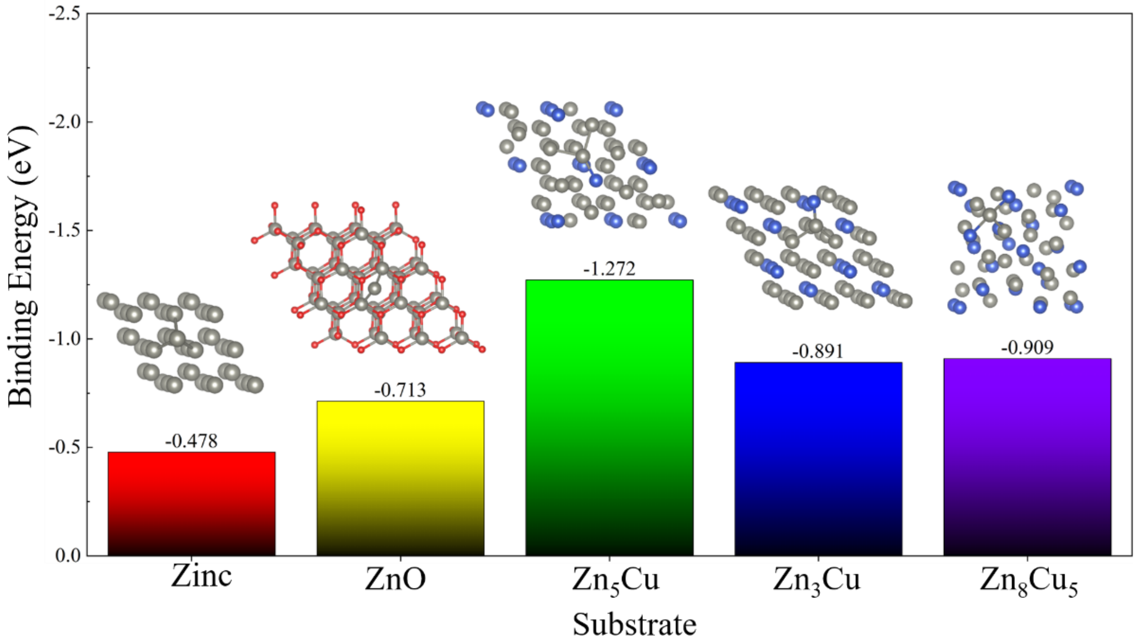

Supplementary Figure 7. The calculated binding energy of Zn on the ZnO and the different Zn-Cu alloy layers. The binding energy of Zn with different substrates was investigated by DFT calculation. Zn has the strongest affinity with the Zn_5Cu (001) facet with a binding energy of -1.272 eV and has the weakest affinity with the Zn (001) (-0.478 eV). The binding energy of Zn with the Zn_3Cu (001) facet (-0.891 eV) and the Zn_8Cu_5 (001) facet (-0.909 eV) seem similar. Obviously, the ZnO (001) facet shows a modest binding ability to Zn with a binding energy of -0.713 eV, stronger than the Zn (001) facet and weaker than the other Cu-Zn alloy facets.

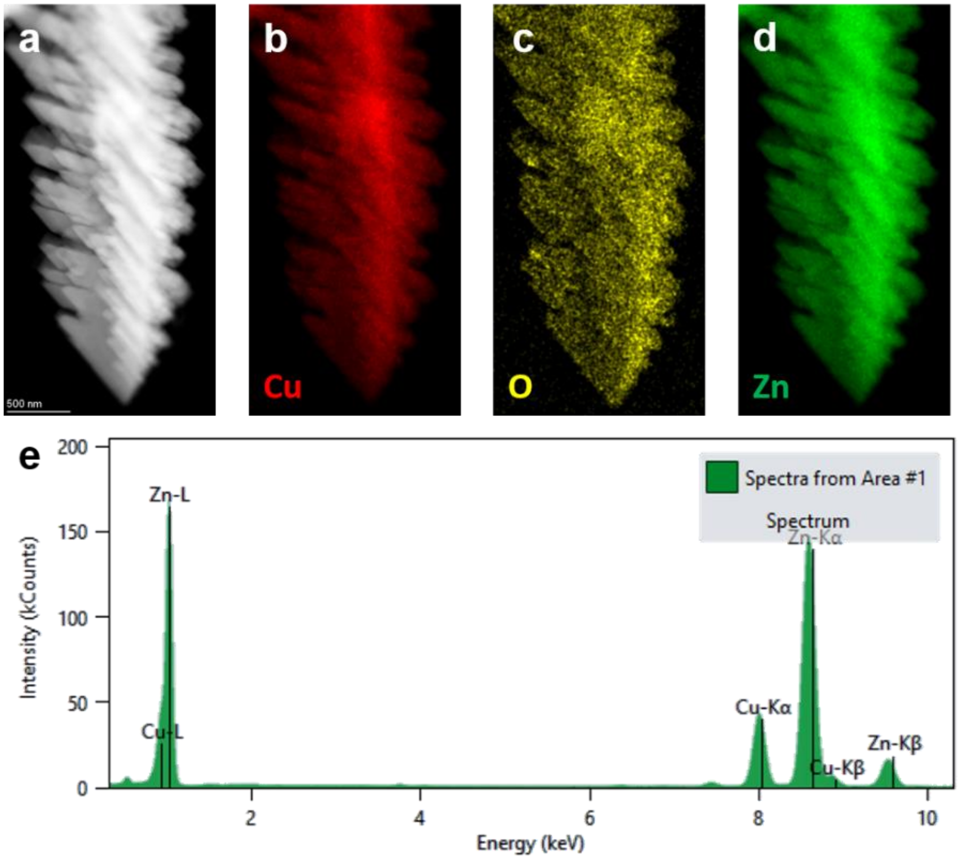

Supplementary Figure 8. EDS mapping and corresponding EDS spectra of Zn-Cu anode.

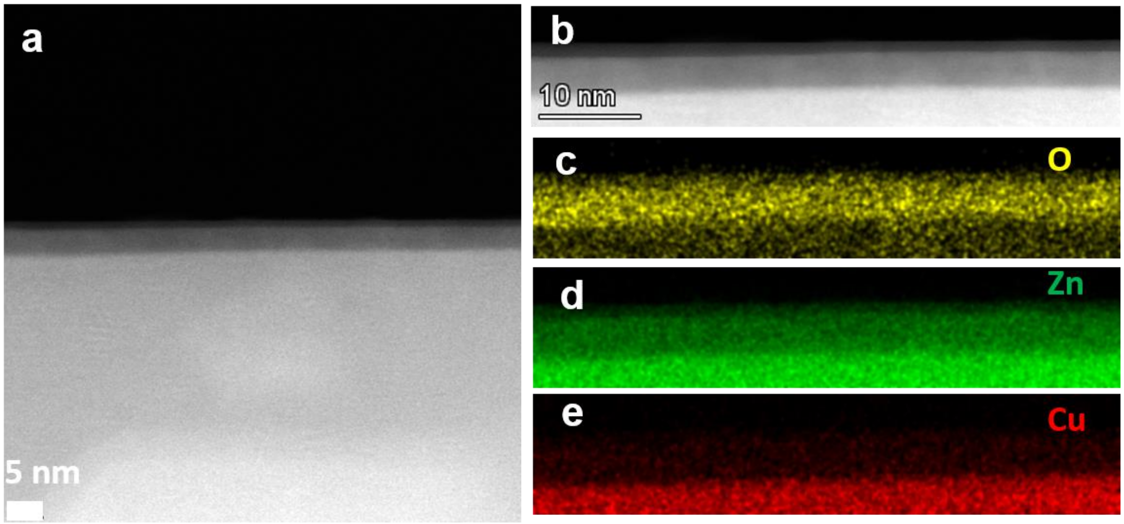

Supplementary Figure 9. EDS mapping of Zn-Cu anode. It can be seen that the terraced branches were covered with a Zn and O-rich layer with straight interfaces

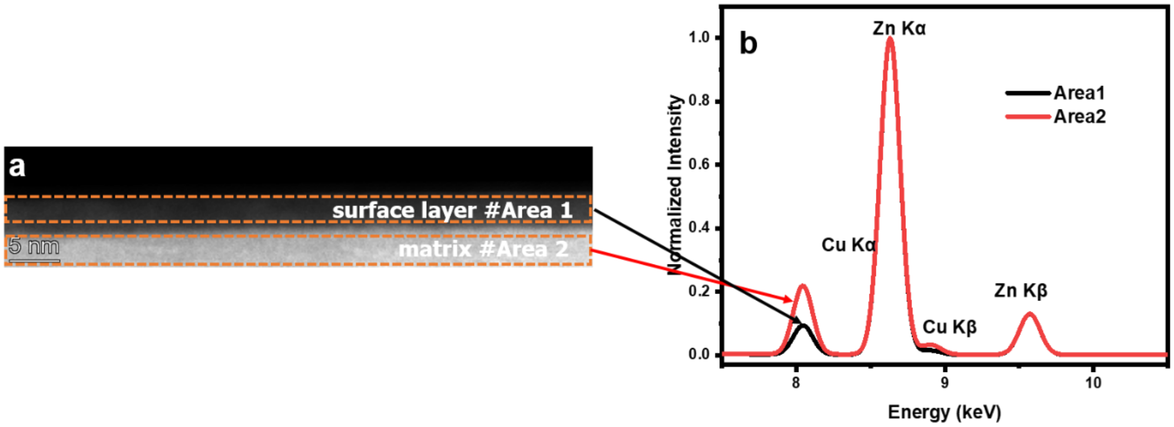

Supplementary Figure 10. (a) HAADF image of EDS analysis and area selection for quantitative analysis. (b) EDS spectrum of the surface and matrix layer, revealing the lower Cu content in the surface.

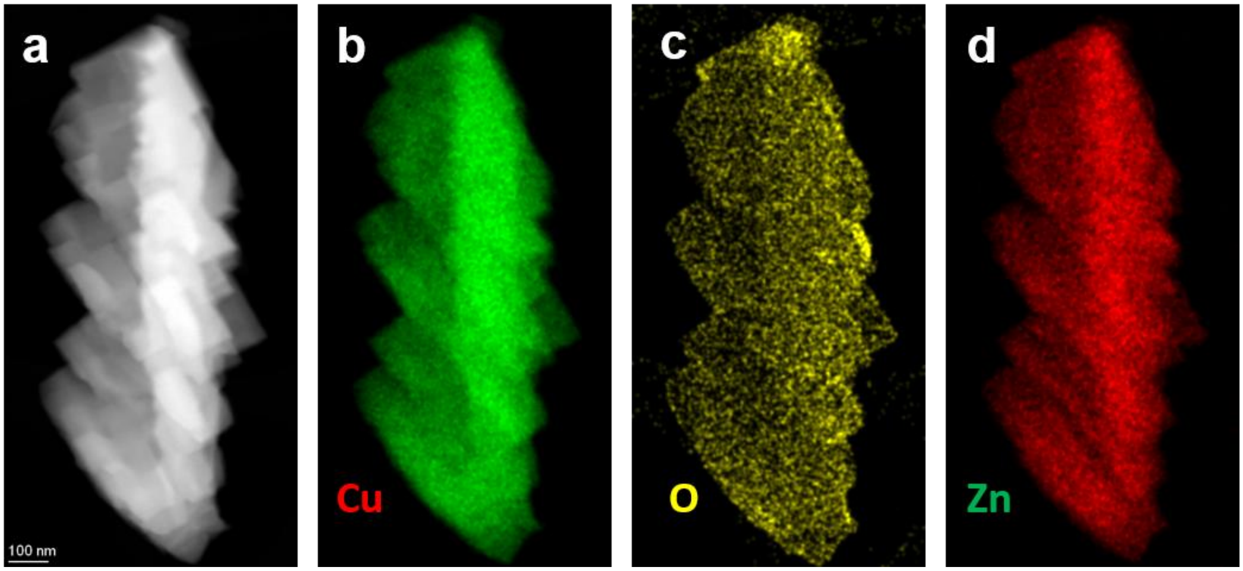

Supplementary Figure 11. EDS mapping of Zn-Zn_5Cu anode after cycling using a single cation-based electrolyte (2 M $ZnSO_4$).

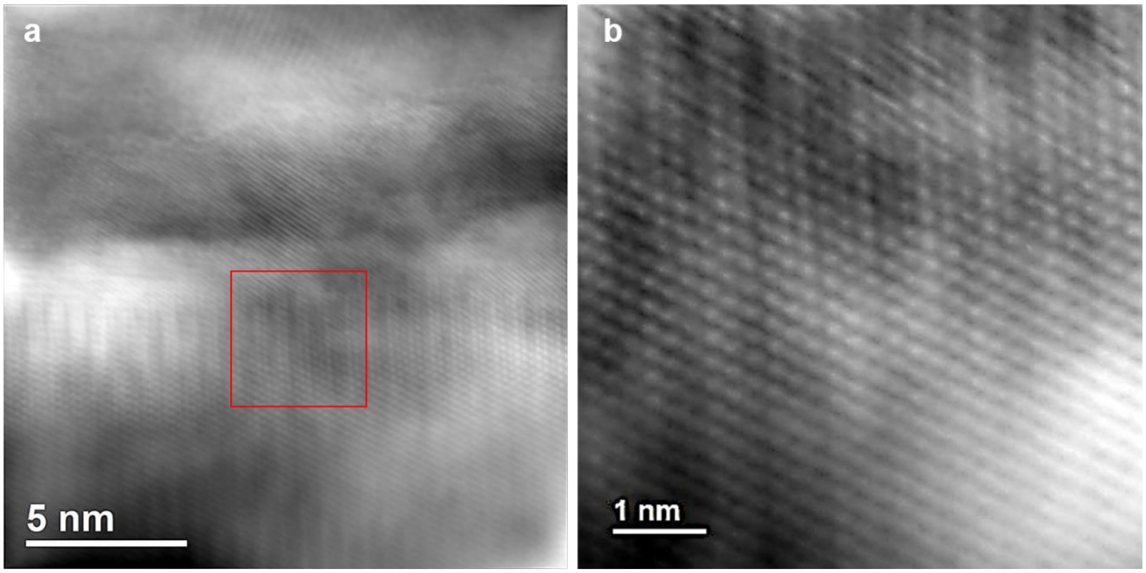

Supplementary Figure 12. (a) Atomic HAADF image of Zn-Cu anode before cycling. (b) Magnified atomic STEM image of red square in (a).

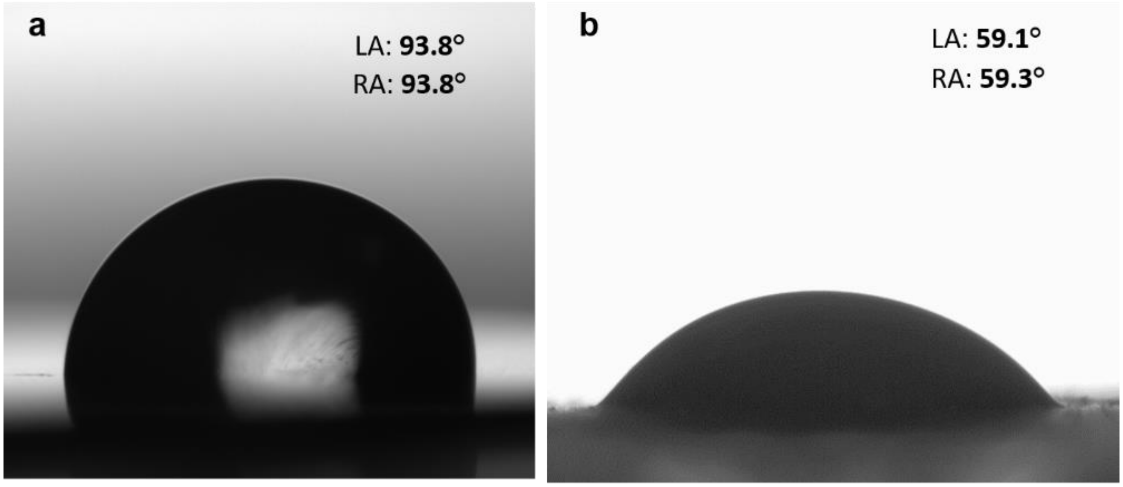

Supplementary Figure 13. Contact angle measurements of (a) the pristine Zn electrode (CA: L: 93.8° R: 93.8°) and (b) the Zn-Cu anode (CA: L: 59.1°; R: 59.3°). On the surface of the Zn-Cu anode, a much smaller contact angle of 59.2 ±0.1^{o} was observed, which could be attributed to the 3D forest-like structure.

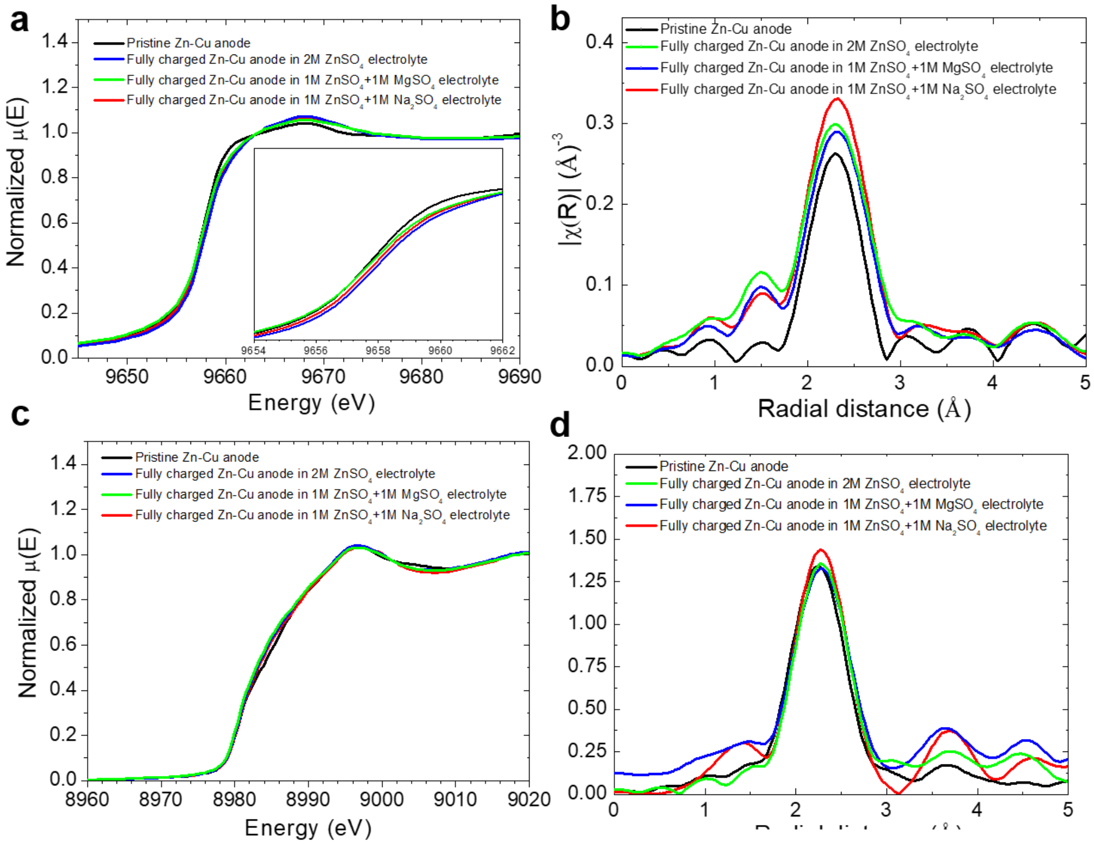

Supplementary Figure 14. Zn K-edge (a) XANES and (b) EXAFS, and Cu K-edge (c) XANES and (d) EXAFS of Zn_5Cu anode at pristine and fully charged states for MnO_2//Zn-Cu batteries in dual-cation electrolytes.

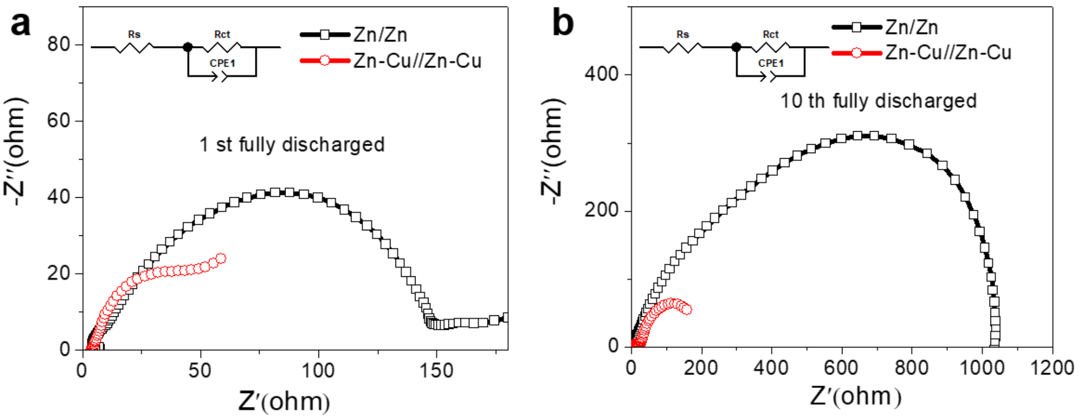

Supplementary Figure 15. Electrochemical impedance spectroscopy (EIS) of the symmetric Zn//Zn and Zn-Cu//Zn-Cu cells after cycling in the Zn-based electrolyte (2 M $ZnSO_4$).

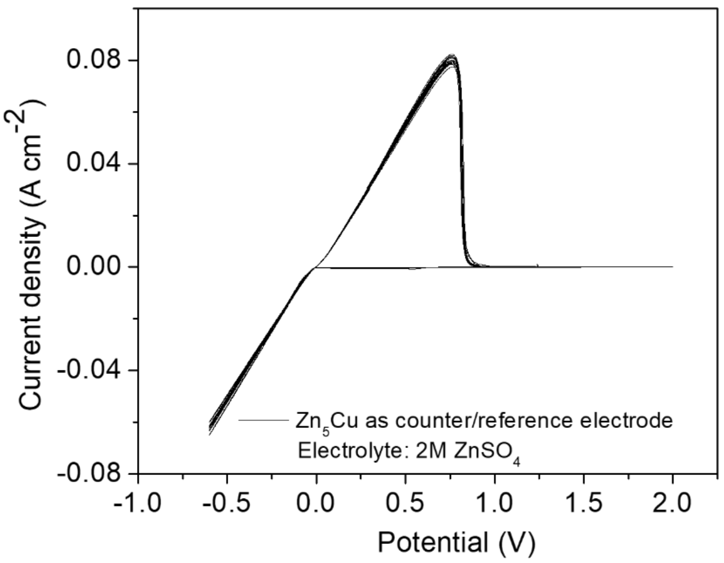

Supplementary Figure 16. Cyclic voltammetry (CV) curves of the single-cation Zn electrolyte for 50 cycles in a range of -0.6-2V. Scan rate: 1 mV s^{-1}; Working electrode: Pt; Reference and counter electrodes: Zn-Cu alloys. Here, we conducted a CV test in a three-electrode set-up to illustrate the deposition/dissolution process and its electrochemical stability behaviors. The high reversibility demonstrates the good stability of Cu-Zn alloy-based anode in the electrolyte 3 (2M $ZnSO_4$).

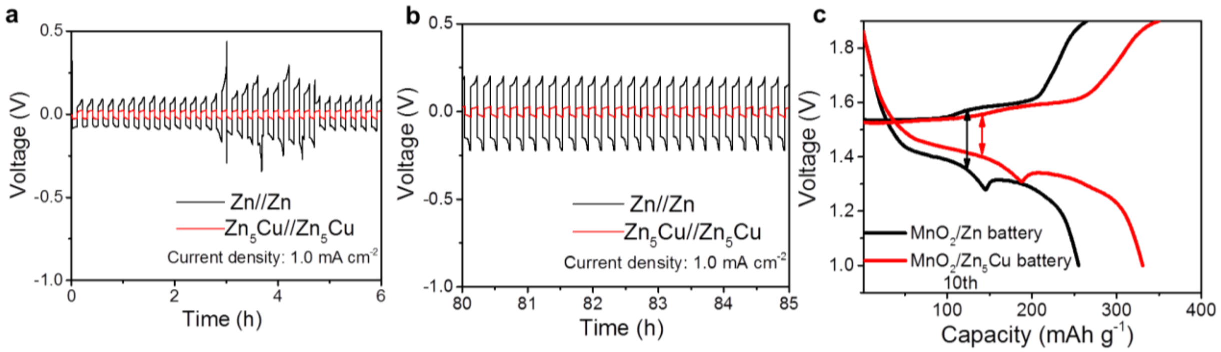

Supplementary Figure 17. (a, b) Enlarged views of voltage-time curves for the symmetric Zn//Zn cells and Zn-Cu//Zn-Cu cells at a current density of 1.0 mA cm^{-2}. (c) Typical charge/discharge curves of aqueous MnO_2//Zn and MnO_2//Zn-Cu batteries.

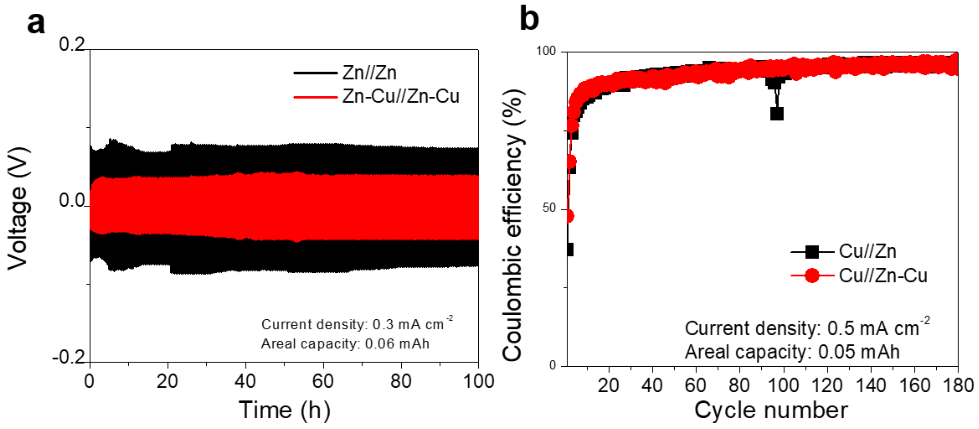

Supplementary Figure 18. (a) Long-term stability of Zn//Zn and Zn-Cu//Zn-Cu cells at a low current density of 0.3 mA cm^{-2} (Areal capacity: 0.06 mAh cm^{-2}). (b) Coulombic efficiencies of Zn plating and stripping at a low current density of 0.5 mA cm^{-2}. Working electrode: Cu. Counter electrode: Zn foil or Zn-Cu. Under a low current density of 0.3 mA cm^{-2} and 0.5 mA cm^{-2}, the Zn-Cu alloy-based anodes show a superior electrochemical cycling performance compared with that of the pristine Zn anode.

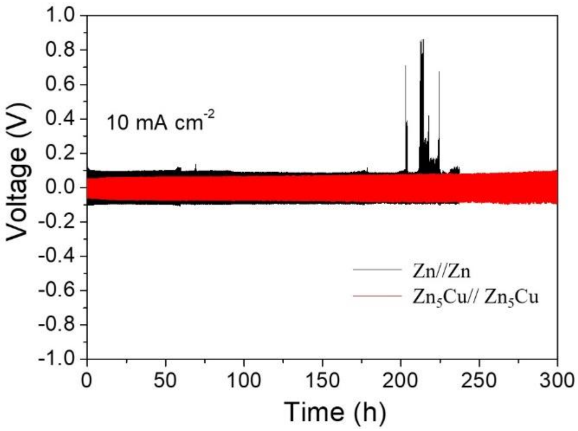

Supplementary Figure 19. Long-term galvanostatic cycling performance of symmetric Zn-Cu and pristine Zn cells at a current density of 10 mA cm^{-2} (Areal capacity: 1 mAh cm^{-2}). The long-term galvanostatic cycling performance of symmetric Zn-Cu and pristine Zn cells at a current density of 10 mA cm^{-2} (areal capacity: 1 mAh cm^{-2}) for over 300 h confirmed that the Zn-Cu anode has a superior electrochemical performance even under higher areal capacity and higher current densities after long-term cycling compared with pristine Zn metal anode.

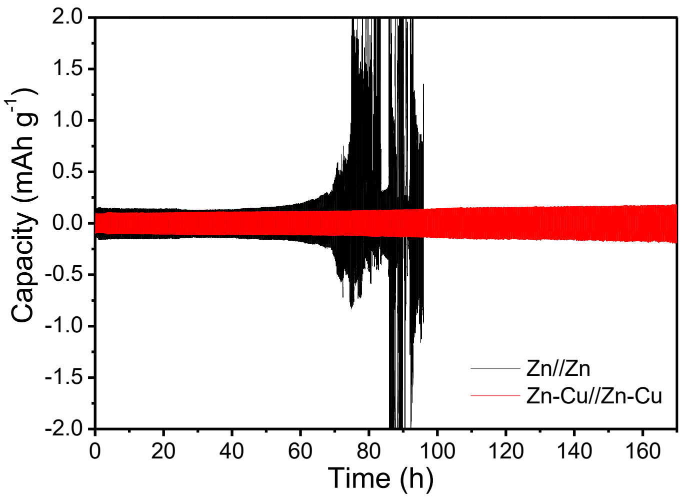

Supplementary Figure 20. Long-term galvanostatic cycling performance of symmetric Zn-Cu and pristine Zn cells at a temperature of 45 °C (Current density of 20 mA cm^{-2}, areal capacity: 2 mAh cm^{-2}).

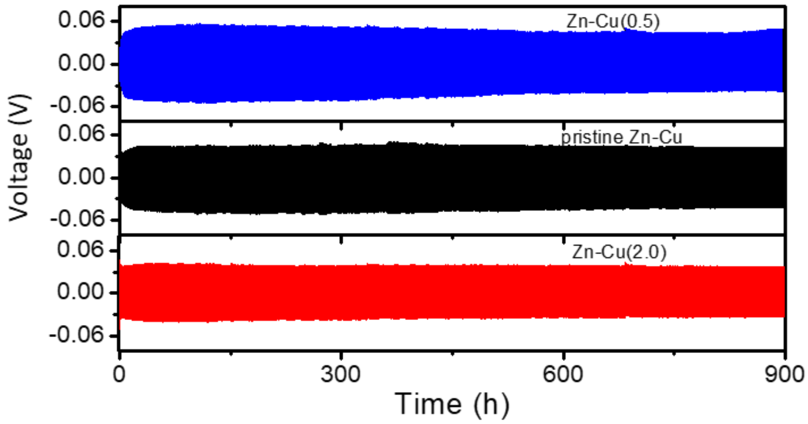

Supplementary Figure 21. Long-term stability of Zn-Cu//Zn-Cu symmetric cells using Zn-Cu anodes prepared from different-content precursors ($CuSO_4$) via the alloy electrodeposition method. The pristine Zn-Cu was chosen as a reference sample. (Current density: 1 mA cm^{-2}; areal capacity: 0.1 mAh cm^{-2}).

We also tested the electrochemical performance of the Zn-Cu alloy with different content of Cu. The symmetric Zn-Cu//Zn-Cu cells show that the Zn-Cu alloy with a higher Cu content has a lower overpotential and also exhibits an excellent cycling performance after long-term (900 h) Zn plating/stripping processes.

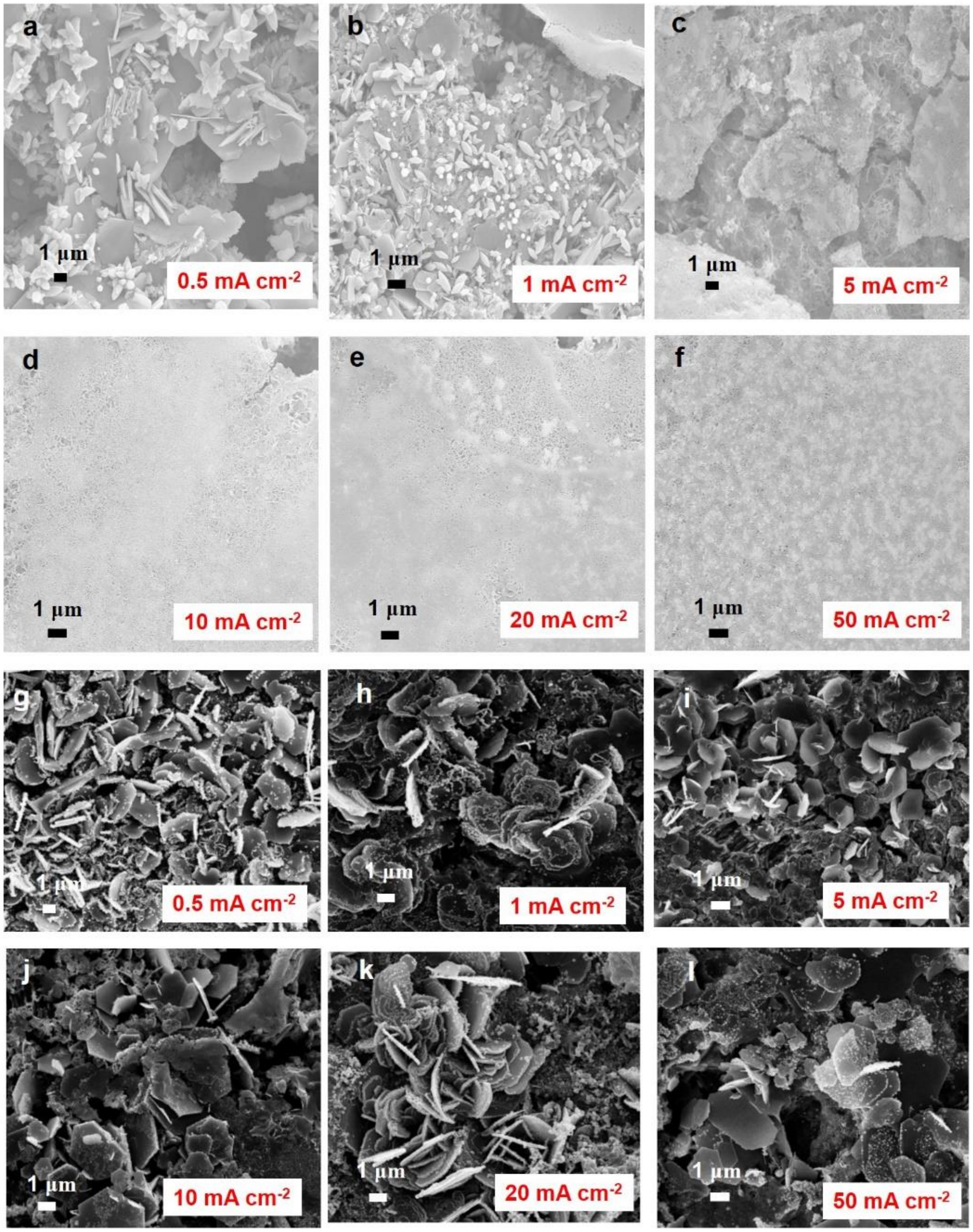

Supplementary Figure 22. Surface SEM images of (a-f) Zn-Cu and (g-l) Zn anodes after Zn plating at different current densities of (a) 0.5, (b) 1, (c) 5, (d) 10, (e) 20, and (f) 50 mA cm^{-2} (Areal capacity: 1.0 mAh cm^{-2}). Different current densities from 1 mA cm^{-2} to 50 mA cm^{-2} were employed to analyze the Zn plating processes by *ex-situ* SEM. A smooth surface of the Zn-Cu anode without dendrite growth after Zn plating can be observed under different current densities from 0.5 mA cm^{-2} to 50 mA cm^{-2}.

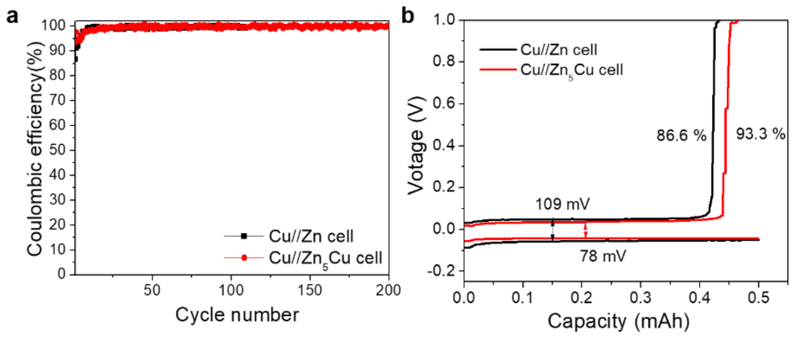

Supplementary Figure 23. Electrochemical performance of Zn-Cu anodes in asymmetric cells. (a) Galvanostatic cycling performance of Cu//Zn and Cu//Zn-Cu cells at a high current density of 5.0 mA cm^{-2} (Areal capacity: 0.5 mAh cm^{-2}). (b) The corresponding plating curves of Cu//Zn and Cu//Zn-Cu cells in the 1st cycle.

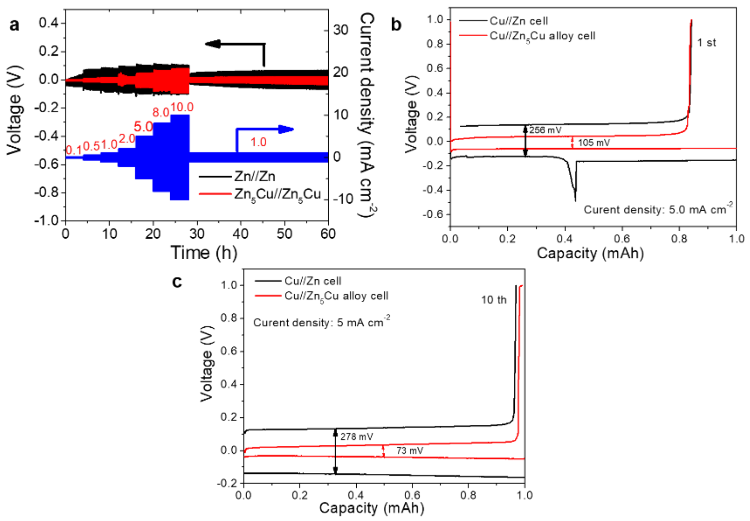

Supplementary Figure 24. Electrochemical performance of Zn-Cu anode in aqueous electrolytes (2 M $ZnSO_4$ + 0.1 M $MnSO_4$). (a) Rate performance of Zn//Zn and Zn-Cu//Zn-Cu cells at different current densities from 0.1 to 10 mA cm^{-2}. (b) 1st cycle and (c) 10th cycle plating/stripping curves of Cu//Zn and Cu//Zn-Cu cells. It shows the Zn plating/stripping profiles under different current densities from 0.1 to 10 mA cm^{-2}. The overpotential of the Zn-Cu//Zn-Cu cell is lower than that of the Zn//Zn cell under different current densities, indicating the excellent rate performance of the Zn-Cu anode. The Zn plating/stripping profiles tested at 5.0 mA cm^{-2} show greatly reduced overpotentials of Cu//Zn-Cu cell (105 mV) compared with that of Cu//Zn cell (256 mV) in the initial cycle.

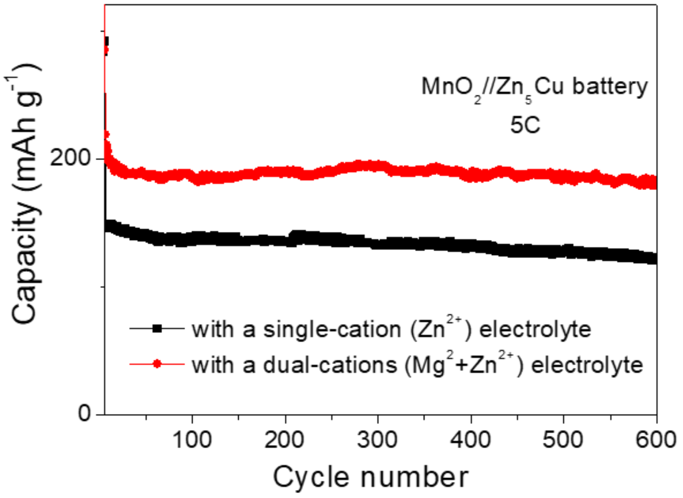

Supplementary Figure 25. Long-term stability of MnO_2//Zn_5Cu cell batteries in a dual-cation electrolyte of "Electrolyte 2" and in a single-cation electrolyte (2M $ZnSO_4$) at 5 C.

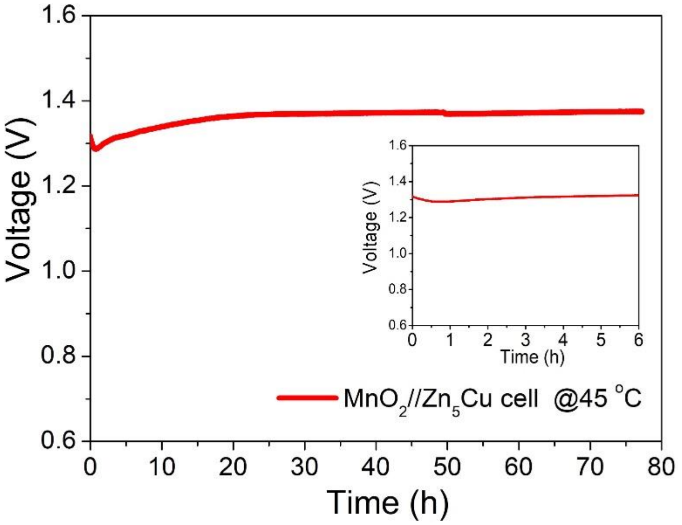

Supplementary Figure 26. Self-discharge test on a MnO_2//Zn_5Cu cell in the Mg^{2+} ion-containing electrolyte (1 M $ZnSO_4$+ 1M $MgSO_4$) at 45 °C. We tested the self-discharge of the Zn-Cu//MnO_2 battery at 45 °C. The open-circuit voltage (OCV) of the Zn-Cu//MnO_2 cell was kept very stable even after over 70 h without any degradation, confirming the stability of the Zn-Cu//MnO_2 battery in the Mg^{2+}-containing dual-cation electrolyte.

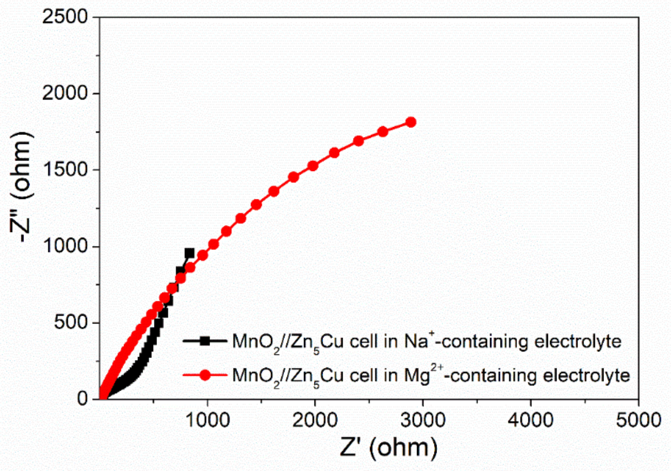

Supplementary Figure 27. Electrochemical impedance spectroscopy (EIS) of MnO_2//Zn_5Cu cells using Mg-containing electrolyte (1 M $ZnSO_4$ + 1 M $MgSO_4$) and Na-containing electrolyte (1 M $ZnSO_4$ + 1 M Na_2SO_4) at a fully discharged state. The improved charge-transfer kinetics can enhance the dynamics of cells in ion storage processes, thus could increase the capacity for dual-cation battery systems using Na-containing electrolytes at a high rate.

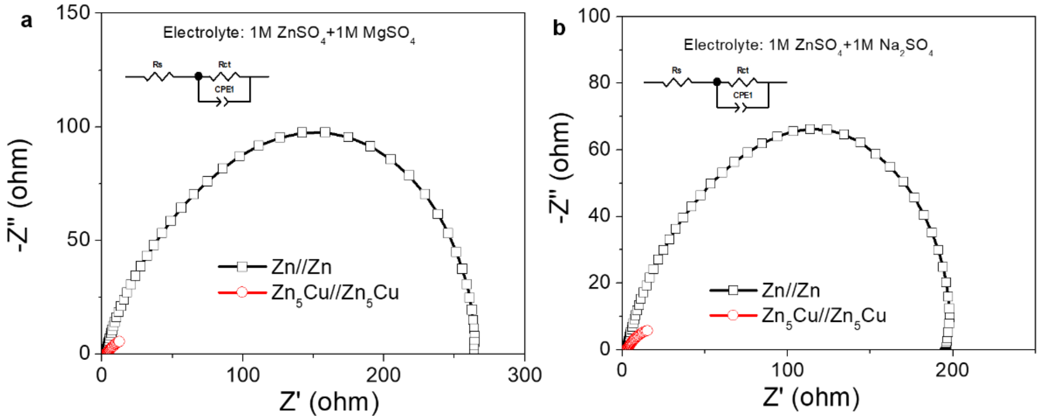

Supplementary Figure 28. Electrochemical impedance spectroscopy (EIS) of Zn//Zn and Zn-Cu//Zn-Cu cells using (a) Mg-containing electrolyte (1 M $ZnSO_4$ + 1 M $MgSO_4$) and (b) Na-containing electrolyte (1 M $ZnSO_4$ + 1 M Na_2SO_4). The charge-transfer kinetics of the Zn-Cu//Zn-Cu cell investigated by EIS demonstrates a remarkably reduced charge-transfer resistance than the Zn//Zn cell in dual-cation electrolytes, confirming the excellent dynamics of the Zn-Cu anode. The enhanced charge-transfer kinetics for aqueous batteries will facilitate the rate performance.

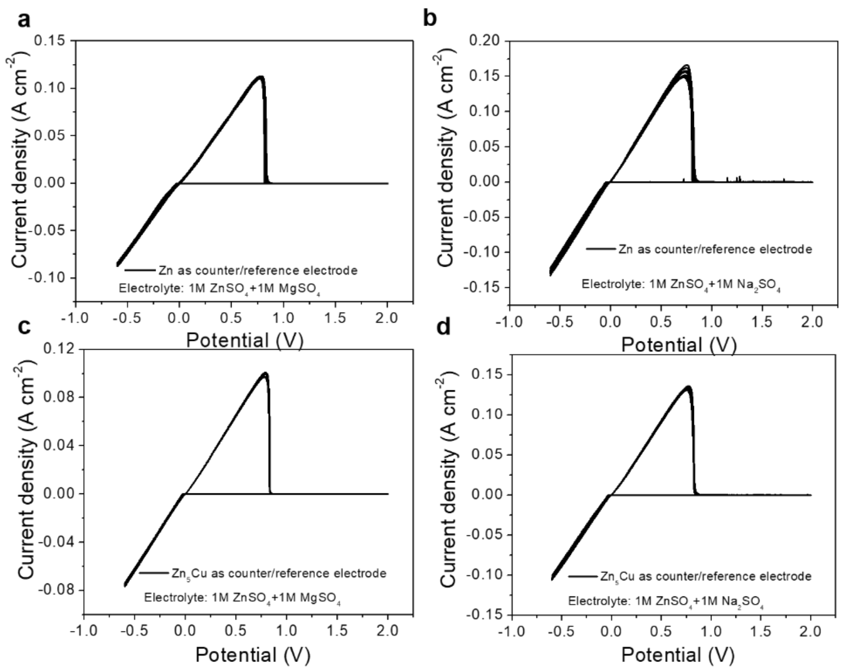

Supplementary Figure 29. Cyclic voltammetry curves of hybrid electrolytes for 15 cycles in a range of -0.6-2V. Scan rate: 1 mV s^{-1}; Working electrode: Pt; Reference and counter electrodes: Zn or Zn-Cu anode. (a, c) Mg-containing electrolyte (1 M $ZnSO_4$ + 1 M $MgSO_4$) and (b, d) Na-containing electrolyte (1 M $ZnSO_4$ + 1 M Na_2SO_4).

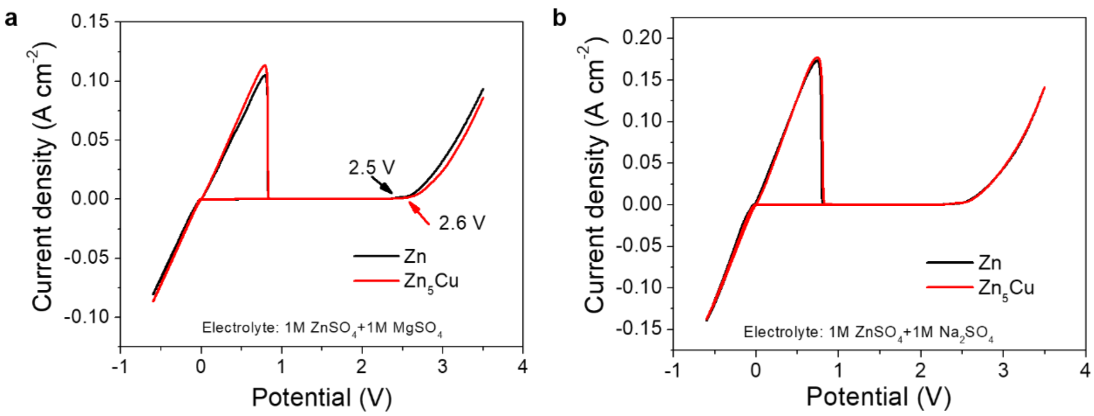

Supplementary Figure 30. Cyclic voltammetry curves of hybrid electrolytes in a range of 0.6-3.5V. Scan rate: 1 mV s^{-1}; Working electrode: Pt; Reference and counter electrodes: Zn or Zn-Cu anode. (a) Mg-containing electrolyte (1 M $ZnSO_4$ + 1 M $MgSO_4$) and (b) Na-containing electrolyte (1 M $ZnSO_4$ + 1 M Na_2SO_4).

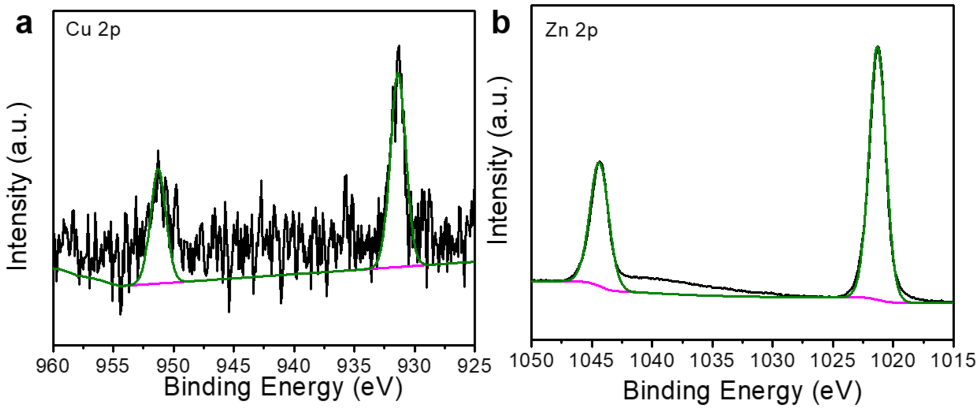

Supplementary Figure 31. X-ray photoelectron spectroscopy (XPS) of as-prepared 3D structured Zn-Cu electrode. It could be observed that the as-prepared sample with Cu (931.3 eV for Cu 2p3/2 and 951.3 eV for Cu 2p1/2) and Zn (1021.3 eV for Zn 2p3/2 and 1044.3 eV for Zn 2p1/2) in both metallic states from XPS.

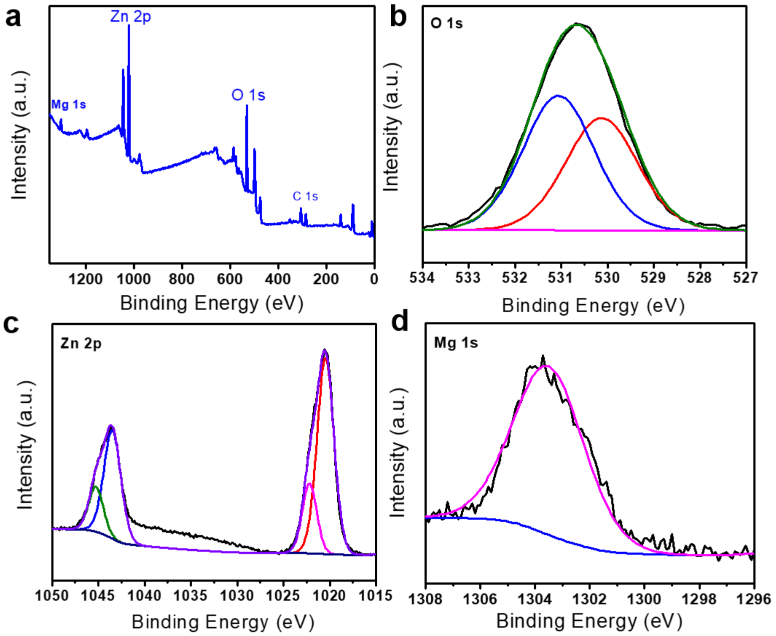

Supplementary Figure 32. XPS spectra of cycled 3D structured Zn-Cu electrode in dual-cation Electrolyte 2 (1 M $ZnSO_4$ + 1 M $MgSO_4$).

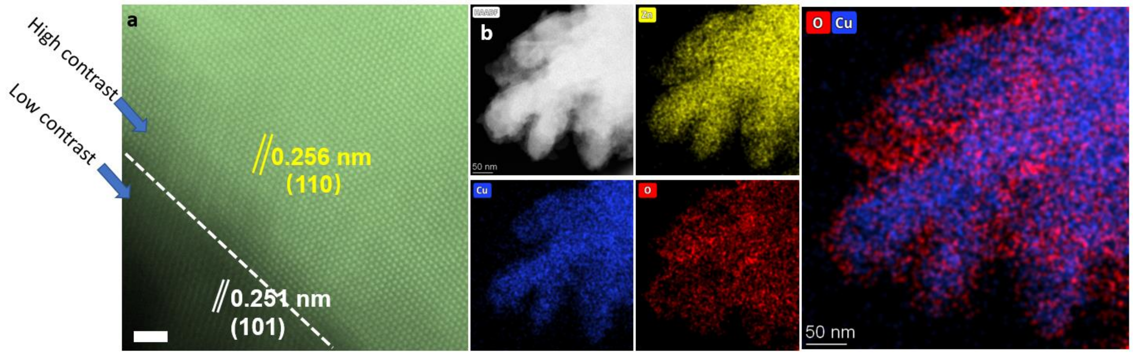

Supplementary Figure 33. (a) Image contrast of ZnO layer and Zn_5Cu core in high-angle annular dark-field scanning transmission electron microscopy (HAADF) image. (b) EDS mapping of O, Zn, and Cu elements of the sample.

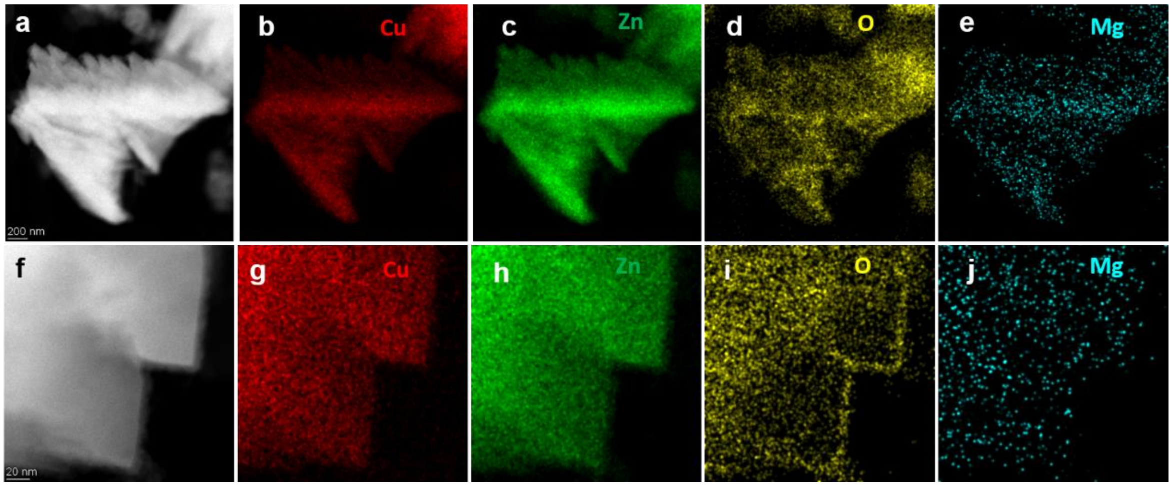

Supplementary Figure 34. EDS mapping of Mg-Zn_5Cu electrode in the different magnification.

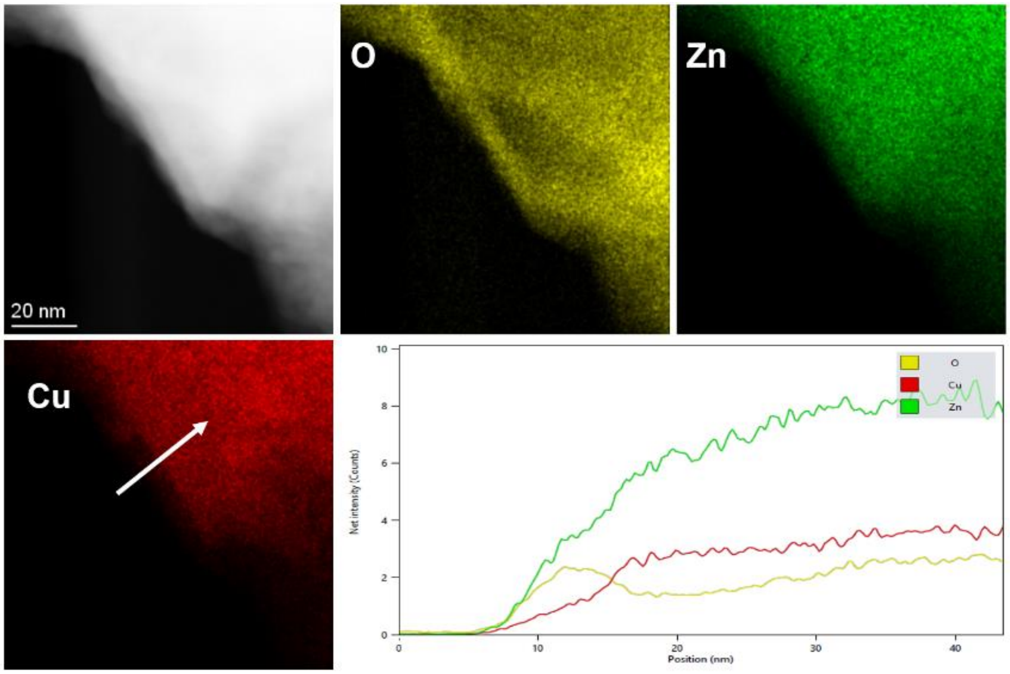

Supplementary Figure 35. EDS mapping of Na-Zn_5Cu electrode after 100 charge/discharge cycles. The corresponding line scan indicates the ZnO layer is still covered on the Na-Zn_5Cu electrode

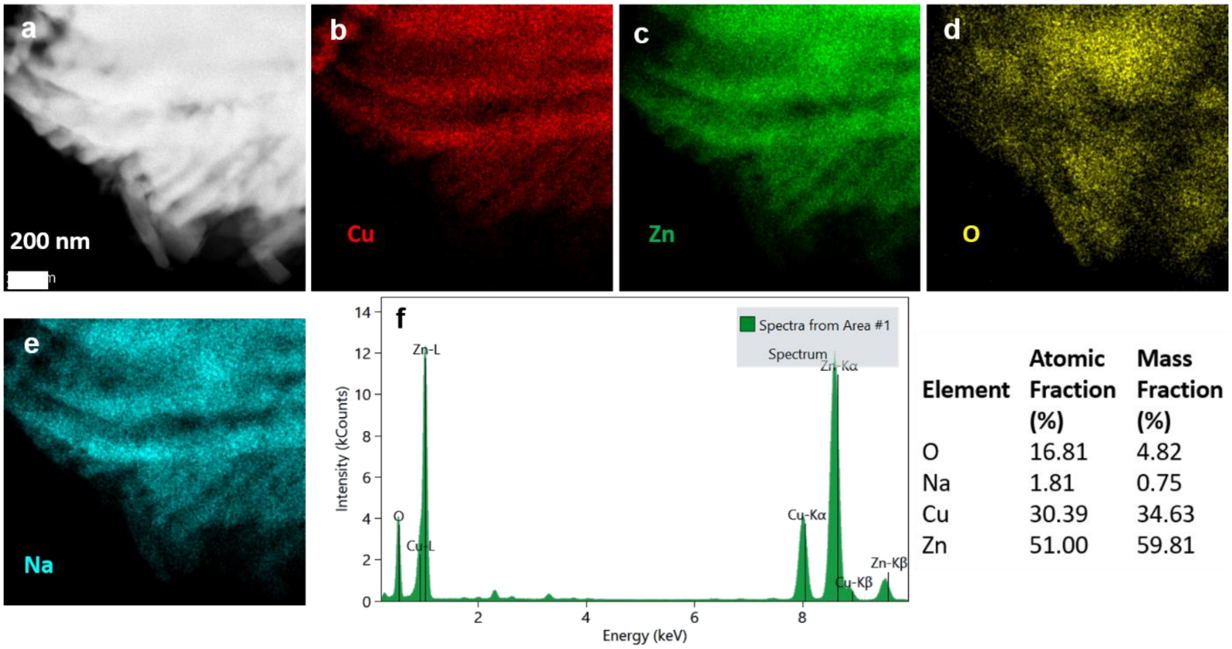

Element	Atomic Fraction (%)	Mass Fraction (%)
O	16.81	4.82
Na	1.81	0.75
Cu	30.39	34.63
Zn	51.00	59.81

Supplementary Figure 36. EDS mapping of the Na-Zn_5Cu electrode.

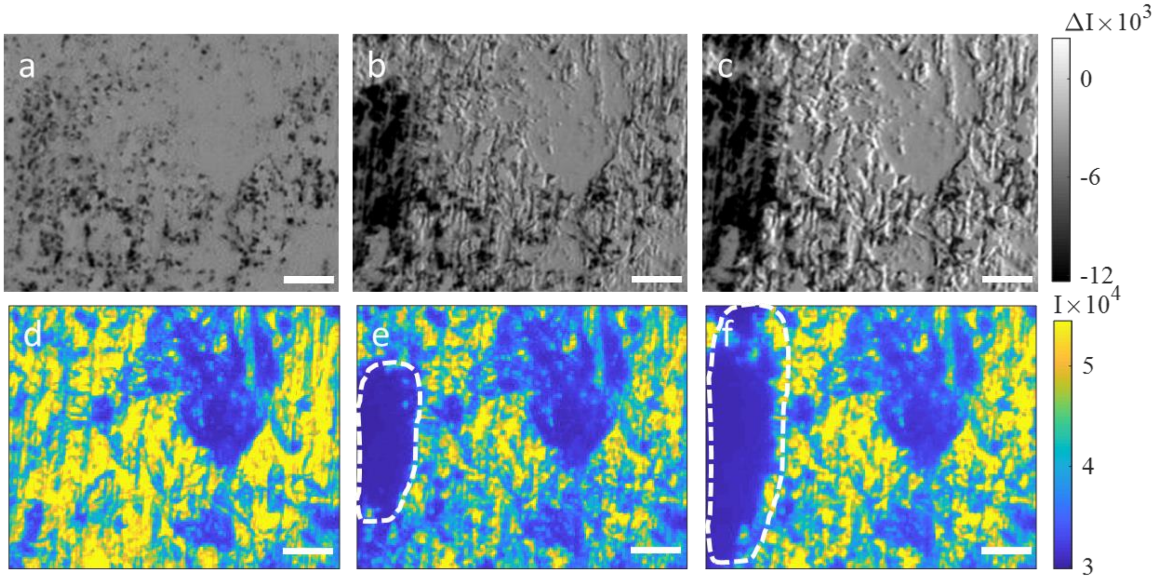

Supplementary Figure 37. *In-situ* visualization of Zn plating on pristine Zn anode**.** (a-c) The differential images of pristine Zn anode during the Zn plating process. The images were taken at 12 s, 240 s, and 720 s of Zn plating and then subtracted from the first image. The contrast shows the intensity change (ΔI) during the deposition. (d-f) Images of the pristine Zn anode at 12 s, 240 s, and 720 s during Zn plating. Current density: 30 mA cm^{-2}; Electrolyte: 1 M $ZnSO_4$ + 1 M $MgSO_4$; Scale bar: 10 μm.

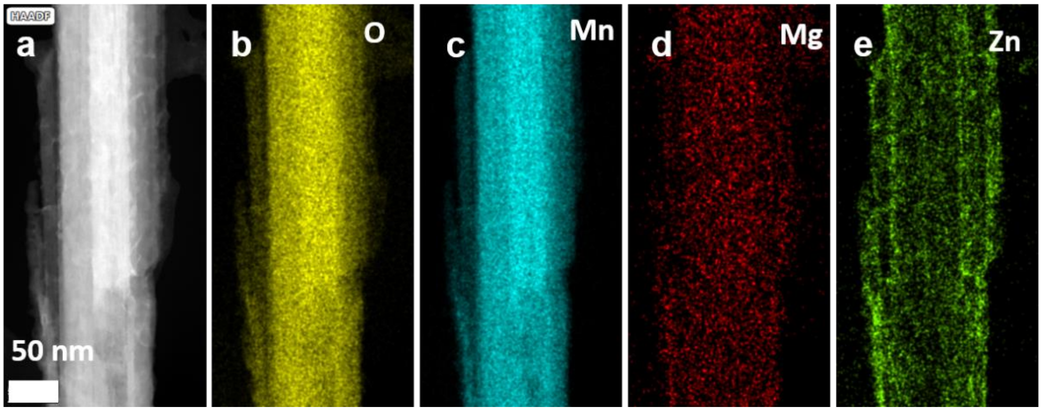

Supplementary Figure 38. EDS mapping of the Mg-MnO_2 electrode.

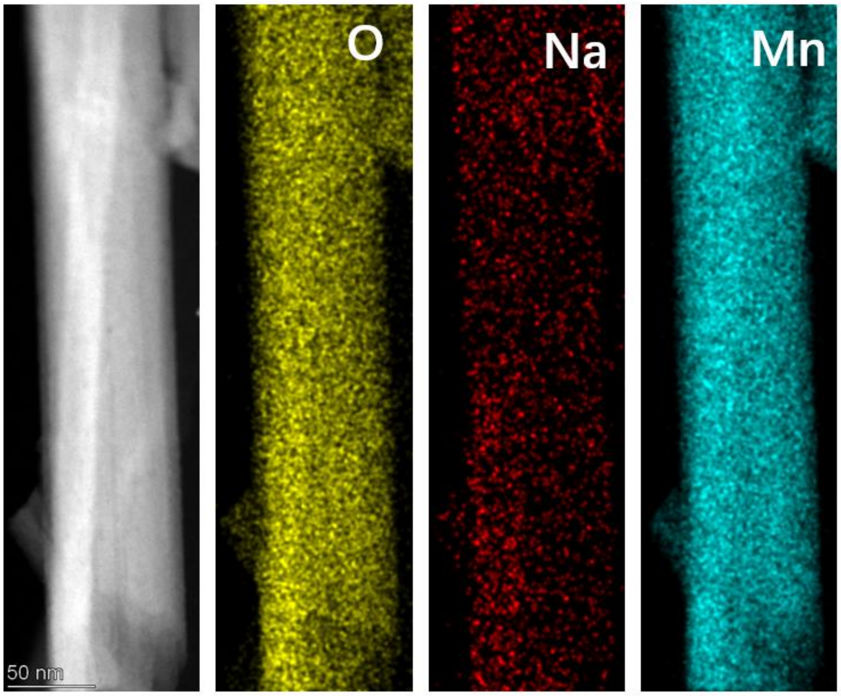

Supplementary Figure 39. EDS mapping of the Na-MnO_2 electrode.

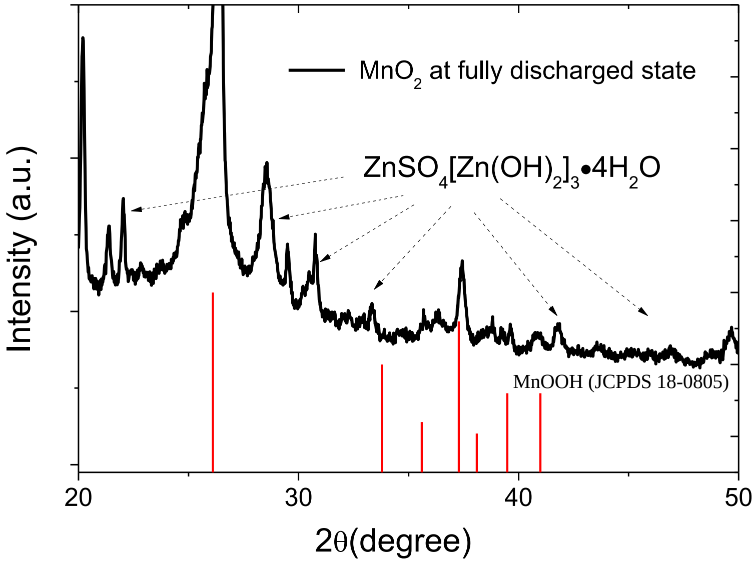

Supplementary Figure 40. XRD pattern of MnO_2 after fully discharged state in the Mg^{2+}-containing dual-cation electrolyte. The XRD pattern of the MnO_2 cathode after full discharge was characterized. The merging characteristic peaks matched well with MnOOH (JCPDS 18–0805) and $ZnSO_4(OH)_6{\cdot}4H_2O$ for cycled MnO_2 cathode at a fully discharged state.

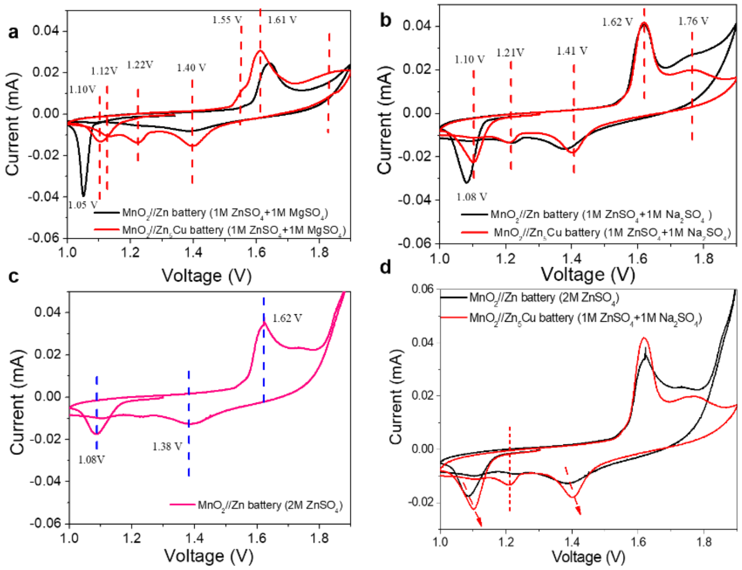

Supplementary Figure 41. CV curves of Zn-Cu//MnO_2 and Zn//MnO_2 batteries using the (a) Mg^{2+}-containing dual-cation electrolyte, (b) Na^{+}-containing dual-cation electrolyte, (c) and (d) traditional electrolyte (2M $ZnSO_4$). In the CV curves, compared with the MnO_2//Zn battery using the 2M $ZnSO_4$ electrolyte and MnO_2//Zn_5Cu battery using the 2M $ZnSO_4$ electrolyte, the MnO_2//Zn_5Cu battery using dual-cation electrolytes have a higher discharging plateau, and also have obvious reduction peaks, which could due to the influence of co-intercalation during discharging process in the Mg^{2+}-containing electrolyte and the Na^{+}-containing electrolyte.

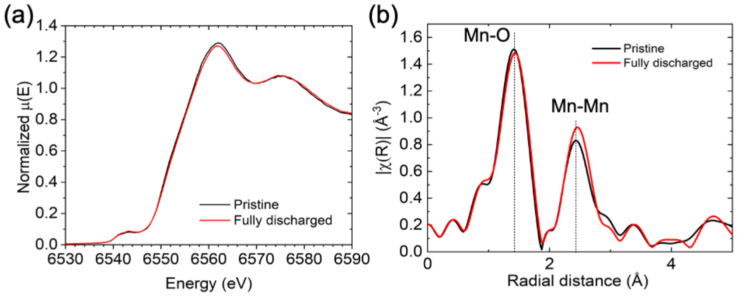

Supplementary Figure 42. Mn K-edge (a) XANES and (b) EXAFS for MnO_2 cathode at pristine and fully charged states in Mg^{2+} containing electrolyte. The XANES region reveals that the bulk MnO_2 structure is preserved after discharging indicated by the close similarity in line shape and position between the two samples. Combined with the results of CV curves, a comparison of MnO_2 cathode EXAFS at pristine and a fully discharged state shows some slight bond distance changes, which could be due to the intercalation of dual cations.

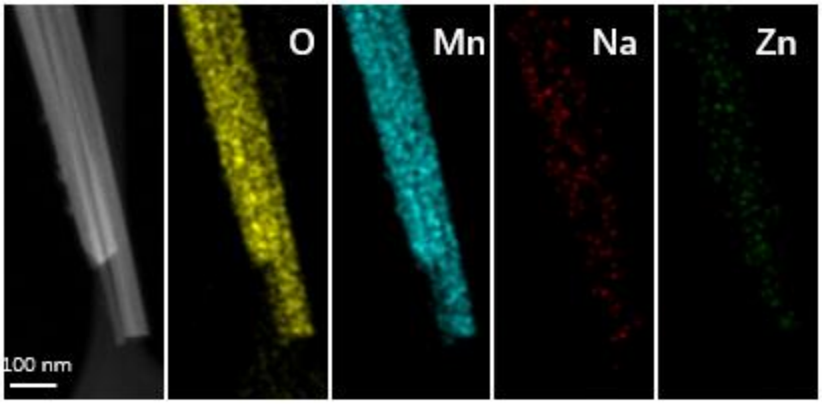

Supplementary Figure 43. EDS mapping of MnO_2 cathode after 100 charge/discharge cycles at the fully charged state in Na^+/Zn^{2+} dual-cation electrolyte (1M $ZnSO_4$+1M Na_2SO_4).

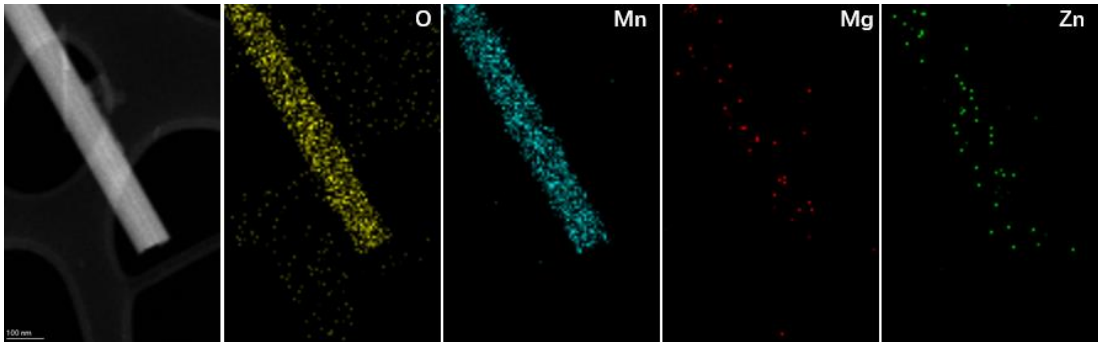

Supplementary Figure 44. EDS mapping of MnO_2 cathode after 100 charge/discharge cycles at the fully charged state in Mg^{2+}/Zn^{2+} dual-cation electrolyte (1M $ZnSO_4$+1M $MgSO_4$).

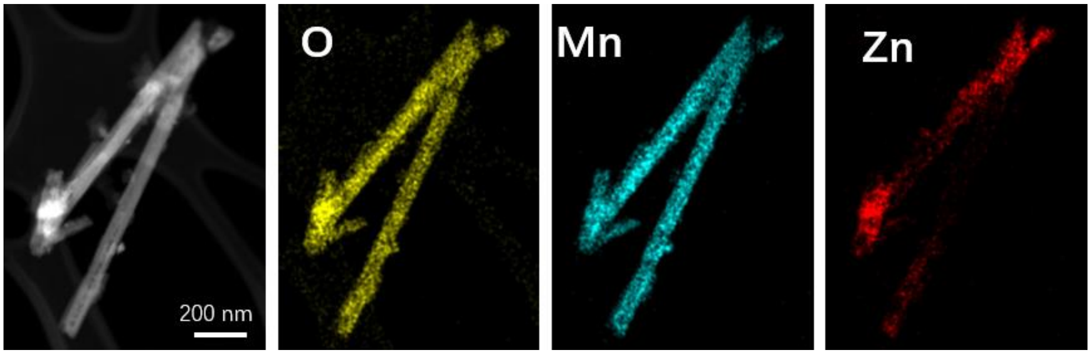

Supplementary Figure 45. EDS mapping of MnO_2 cathode after 100 charge/discharge cycles at the fully charged state in a single-cation electrolyte (2M $ZnSO_4$).

References

1. Xu C, Li B, Du H, Kang F. Energetic Zinc Ion Chemistry: The Rechargeable Zinc Ion Battery. *Angew Chem Int Edit* **51**, 933-935 (2012).

2. Nam KW, Kim H, Choi JH, Choi JW. Crystal water for high performance layered manganese oxide cathodes in aqueous rechargeable zinc batteries. *Energy Environ Sci* **12**, 1999-2009 (2019).

3. Xiong T, *et al.* Defect Engineering of Oxygen-Deficient Manganese Oxide to Achieve High-Performing Aqueous Zinc Ion Battery. *Adv Energy Mater* **9**, 1803815 (2019).

4. Alfaruqi MH, *et al.* Electrochemically Induced Structural Transformation in a gamma-MnO_2 Cathode of a High Capacity Zinc-Ion Battery System. *Chem Mater* **27**, 3609-3620 (2015).

5. Alfaruqi MH, *et al.* A layered delta-MnO_2 nanoflake cathode with high zinc-storage capacities for eco-friendly battery applications. *Electrochem Commun* **60**, 121-125 (2015).

6. Zeng Y, *et al.* Achieving Ultrahigh Energy Density and Long Durability in a Flexible Rechargeable Quasi-Solid-State Zn-MnO_2 Battery. *Adv Mater* **29**, 1700274 (2017).

7. Zhang N, *et al.* Rechargeable aqueous zinc-manganese dioxide batteries with high energy and power densities. *Nat Commun* **8**, 405 (2017).

8. Alfaruqi MH, *et al.* Electrochemical Zinc Intercalation in Lithium Vanadium Oxide: A High-Capacity Zinc-Ion Battery Cathode. *Chem Mater* **29**, 1684-1694 (2017).

9. Hu P, *et al.* Zn/V_2O_5 Aqueous Hybrid-Ion Battery with High Voltage Platform and Long Cycle Life. *ACS Appl Mater Inter* **9**, 42717-42722 (2017).

10. Ming F, Liang H, Lei Y, Kandambeth S, Eddaoudi M, Alshareef HN. Layered $Mg_xV_2O_5 \cdot nH_2O$ as Cathode Material for High-Performance Aqueous Zinc Ion Batteries. *ACS Energy Letters* **3**, 2602-2609 (2018).

11. He P, *et al.* Layered VS_2 Nanosheet-Based Aqueous Zn Ion Battery Cathode. *Adv Energy Mater* **7**, 1601920 (2017).

12. Zhang L, Chen L, Zhou X, Liu Z. Towards High-Voltage Aqueous Metal-Ion Batteries Beyond 1.5 V: The Zinc/Zinc Hexacyanoferrate System. *Adv Energy Mater* **5**, (2015).

13. Zhang N, *et al.* Cation-Deficient Spinel $ZnMn_2O_4$ Cathode in $Zn(CF_3SO_3)_2$ Electrolyte for Rechargeable Aqueous Zn-Ion Battery. *J Am Chem Soc* **138**, 12894-12901 (2016).

14. He P, *et al.* Sodium Ion Stabilized Vanadium Oxide Nanowire Cathode for High-Performance Zinc-Ion Batteries. *Adv Energy Mater* **8**, 1702463 (2018).

15. Soundharrajan V, *et al.* $Na_2V_6O_{16}\cdot 3H_2O$ Barnesite Nanorod: An Open Door to Display a Stable and High Energy for Aqueous Rechargeable Zn-Ion Batteries as Cathodes. *Nano Lett* **18**, 2402-2410 (2018).
