# Supplementary material for: Three-dimensional Zn-based alloys for dendrite-free aqueous Zn battery in dual-cation electrolytes
Source: Nat Commun. 2022 Dec 23;13:7922. doi: 10.1038/s41467-022-35618-2 (PMC9789050; doi:10.1038/s41467-022-35618-2)
Supplement: Supplementary file 2 — Description of Additional Supplementary Files [file 41467_2022_35618_MOESM2_ESM.pdf]

Description of Additional Supplementary Files

File Name: Supplementary Movie 1
Description: **Description: Dendrite growth on the pristine Zn surface.** The video was recorded with a 20X water immersion objective and 26 frames per second. The experiment was performed at a constant current density of 30 mA cm^{-2} for 720s.

File Name: Supplementary Movie 2
Description: **Description: Differential optical video.** The video was recorded with a 20X water immersion objective and 5 frames per second under a constant current density of 50 mA cm^{-2} for the 1200s, obtained by subtracting the first frame.

File Name: Supplementary Movie 3
Description: **Description: Zn plating process in electrolyte 2.** The video was recorded with a 20X water immersion objective and 5 frames per second under a constant current density of 50 mA cm^{-2} for the 1200s. The plating process in electrolyte 2 was repeated 5 times to demonstrate the morphology change.

File Name: Supplementary Movie 4
Description: **Zn stripping process in electrolyte 2.** The video was recorded with a 20X water immersion objective and 5 frames per second under a constant current density of 50 mA cm^{-2} for the 1200s. The stripping process in electrolyte 2 was repeated 5 times to demonstrate the morphology change.

File Name: Supplementary Movie 5
Description: **Zn plating process in electrolyte 1.** The video was recorded with a 20X water immersion objective and 5 frames per second under a constant current density of 50 mA cm^{-2} for 600s. The plating process in electrolyte 1 was repeated 5 times to demonstrate the morphology change.

File Name: Supplementary Movie 6
Description: **Zn stripping process in electrolyte 1.** The video was recorded with a 20X water immersion objective and 5 frames per second under a constant current density of 50 mA cm^{-2} for 900s. The stripping process in electrolyte 1 was repeated 5 times to demonstrate the morphology change.

File Name: Supplementary Movie 7
Description: **Small current density Zn plating process.** The plating was performed in a single cation Zn electrolyte (2M $ZnSO_4$ in DI water) under the current density of 5 mA cm^{-2} for 3000s and the video was recorded with a 20X water immersion. The entire process was repeated 5 times to better demonstrate the morphology change.

File Name: Supplementary Movie 8
Description: **Small current density Zn stripping process.** The stripping was performed in 2M $ZnSO_4$ under the current density of 5 mA cm^{-2} for 3000s and the video was recorded with a 20X water immersion objective. The stripping process was repeated 5 times to exhibit the morphology change.
